# Directly reprogrammed fragile X syndrome dorsal forebrain precursor cells generate cortical neurons exhibiting impaired neuronal maturation

**DOI:** 10.3389/fncel.2023.1254412

**Published:** 2023-09-21

**Authors:** Nicole Edwards, Catharina Combrinck, Amy McCaughey-Chapman, Bronwen Connor

**Affiliations:** Department of Pharmacology and Clinical Pharmacology, Centre for Brain Research, School of Medical Science, Faculty of Medical and Health Sciences, University of Auckland, Auckland, New Zealand

**Keywords:** fragile X syndrome, direct reprogramming, neurodevelopment, dorsal progenitor, cortical neuron, methylation

## Abstract

**Introduction:**

The neurodevelopmental disorder fragile X syndrome (FXS) is the most common monogenic cause of intellectual disability associated with autism spectrum disorder. Inaccessibility to developing human brain cells is a major barrier to studying FXS. Direct-to-neural precursor reprogramming provides a unique platform to investigate the developmental profile of FXS-associated phenotypes throughout neural precursor and neuron generation, at a temporal resolution not afforded by post-mortem tissue and in a patient-specific context not represented in rodent models. Direct reprogramming also circumvents the protracted culture times and low efficiency of current induced pluripotent stem cell strategies.

**Methods:**

We have developed a chemically modified mRNA (cmRNA) -based direct reprogramming protocol to generate dorsal forebrain precursors (hiDFPs) from FXS patient-derived fibroblasts, with subsequent differentiation to glutamatergic cortical neurons and astrocytes.

**Results:**

We observed differential expression of mature neuronal markers suggesting impaired neuronal development and maturation in FXS- hiDFP-derived neurons compared to controls. FXS- hiDFP-derived cortical neurons exhibited dendritic growth and arborization deficits characterized by reduced neurite length and branching consistent with impaired neuronal maturation. Furthermore, FXS- hiDFP-derived neurons exhibited a significant decrease in the density of pre- and post- synaptic proteins and reduced glutamate-induced calcium activity, suggesting impaired excitatory synapse development and functional maturation. We also observed a reduced yield of FXS- hiDFP-derived neurons with a significant increase in FXS-affected astrocytes.

**Discussion:**

This study represents the first reported derivation of FXS-affected cortical neurons following direct reprogramming of patient fibroblasts to dorsal forebrain precursors and subsequently neurons that recapitulate the key molecular hallmarks of FXS as it occurs in human tissue. We propose that direct to hiDFP reprogramming provides a unique platform for further study into the pathogenesis of FXS as well as the identification and screening of new drug targets for the treatment of FXS.

## 1. Introduction

Fragile X syndrome (FXS) is the most common heritable form of cognitive impairment in humans and the leading monogenic cause of intellectual disability and autism spectrum disorder (ASD), affecting approximately 1:4,000 males and 1:11,000 females (Bagni and Oostra, 2013; Hunter et al., 2014). The molecular cause of FXS arises from loss-of-function mutations in the X-chromosome gene, *FMR1*. Gene silencing is thought to occur early in embryogenesis (~10–11 gestation; Willemsen et al., 2002; Hecht et al., 2017). In nearly all cases, the observed mutation is an expansion of a CGG trinucleotide repeat in the promoter region of the gene (Verkerk et al., 1991). In unaffected individuals, the CGG region is repeated 5 to 54 times. Individuals harboring between 55 and 200 CGG repeats are defined as premutation carriers, and the full mutation state is defined as greater than 200 CGG repeats (Nolin et al., 2019). At this size, hypermethylation of the repeat region leads to the transcriptional silencing of the *FMR1* gene and loss of the protein product of *FMR1*, Fragile X messenger ribonucleoprotein (FMRP). While it is widely accepted that FXS results from the loss or significant reduction in the function of FMRP, knowledge regarding the early molecular mechanisms that link the loss of FMRP to the neuropathology and cognitive impairments that characterize FXS is currently limited (Richter and Zhao, 2021; Bruford, 2022). As a result, mechanism-based disease modifying therapies remain elusive, with few therapeutic agents progressing to phase III clinical trial. Currently, pharmacological treatment for FXS is limited to symptomatic relief of behavioral comorbidities.

In addition to the two major pathogenic allele variants (Full; PM), mitotic instability during neurodevelopment frequently results in somatic cell mosaicism both in CGG repeat size and methylation status of the *FMR1* allele. Mosaicism is known to modify phenotypic outcomes in FXS and is linked to a reduction in FXS severity (Hagerman et al., 1994; Loesch et al., 2012; Pretto et al., 2014) and the development of the late onset neurodegenerative disorder, Fragile X tremor ataxia syndrome (Hwang et al., 2016). The heterogenous mechanisms and phenotypes observed in FXS-associated disorders, reinforces the importance of developing model systems that allow insight into the contribution of variable mutant *FMR1* alleles to phenotypic outcomes. Given the poor translation of preclinical FXS model systems towards clinically actionable targets to date, the development of tractable and representative models of human neurodevelopmental disorders is crucial to our ability to draw physiologically relevant conclusions about the mechanisms of FXS pathogenesis during early neurodevelopment.

FXS patient-derived post-mortem tissue has provided valuable insights on the impact of FMRP loss in human neurons but cannot recapitulate developmental processes (Hinton et al., 1991; Wisniewski et al., 1991; Irwin et al., 2001). Therefore, FXS patient-derived post-mortem tissue does not provide the ability to study the mechanisms contributing to dysregulated neurogenesis throughout development. Our current understanding of the mechanistic basis of FXS progression during development have, until recently, been largely derived from either *FMR1* knock out (The Dutch-Belgian Fragile X Consortium, 1994) or CGG knock in mouse models (Bontekoe et al., 2001). Although an invaluable resource for drug screening and evaluating phenotype development within the *in vivo* niche, mouse models of FXS do not recapitulate the molecular hallmarks of the FXS mutation as they occur in humans, in particular hyper-methylation of the *FMR1* promoter (Brouwer et al., 2007).

It is therefore necessary to develop and utilize neural cell culture models to investigate how the loss of FMRP manifests itself during human neurogenesis. FXS affected human embryonic stem cell (hESC) lines and induced pluripotent stem cells (iPSCs) have provided initial insights into the molecular and cellular changes induced through the loss of FMRP in human neurons (Urbach et al., 2010; Sheridan et al., 2011; Bar-Nur et al., 2012; Doers et al., 2014; Esch et al., 2014; Halevy et al., 2015; Kaufmann et al., 2015). One key finding was that methylation-mediated silencing of the *FMR1* gene and the heterochromatinization of the gene promoter were shown to be developmentally dependent (Eiges et al., 2007). The precise developmental timing and mechanism of gene silencing has yet to be fully elucidated, and it is likely that greater variation in the population exists than previously understood. However, evidence suggests that *FMR1* is expressed in FXS hESCs, and gene silencing occurs upon differentiation. In contrast, FXS patient-derived hiPSCs exhibit silencing of the *FMR1* gene in both the pluripotent and the differentiated states (Urbach et al., 2010) indicating that FXS patient-derived iPSCs cannot recapitulate the developmentally dependent silencing of *FMR1*. Premature gene silencing brings into question the validity of FXS hiPSC cell lines in recapitulating the effect of FMRP loss during neural development.

Direct cell reprogramming offers an alternative and efficient means to derive a self-renewing and highly expandable source of multipotent human induced neural precursor cells (hiNPC) with the potential to differentiate to mature neurons and glia *in vitro* while bypassing pluripotency. Many hiNPC reprogramming protocols employ integrative viral vector delivery systems with a concomitant risk of insertional mutagenesis and enhanced genotoxicity. Alternatively, we have demonstrated the ability to directly generate hiNPCs from adult human dermal fibroblasts using chemically modified mRNA (cmRNA) for the neural genes *SOX2* and *PAX6* (Connor et al., 2018). Furthermore, we recently reported the ability to specifically generate human induced dorsal forebrain precursor cells (hiDFP) using cmRNA *SOX2* and *PAX6* direct reprogramming with subsequent differentiation to functionally mature glutamatergic neurons (Edwards et al., 2021). We will utilize our direct reprogramming technology to generate, for the first time, hiDFPs from FXS patient-derived fibroblasts. We propose that utilization of a direct-to-hiDFP protocol to study FXS will provide a more robust mechanism than the current use of hiPSCs by negating concerns regarding the premature inactivation of the *FMR1* gene as seen in FXS patient-derived hiPSC lines (Bhattacharyya and Zhao, 2015). This study represents a novel approach to develop a robust and efficient human-based cell model of FXS.

## 2. Methods

### 2.1. Human dermal fibroblast direct-to-induced dorsal forebrain precursor cell reprogramming and differentiation

Fibroblasts from clinically diagnosed Fragile X syndrome (FXS) male individuals were sourced from Coriell Institute for Medical Research, with (CGG)n FXS affected status confirmed by Coriell as determined by Southern analysis (Tables 1, 2). Specific repeat lengths however, were not available. Therefore, the CGG lengths for FXS cell lines used are listed as reported in other studies based on the untransformed source fibroblasts. Control lines were sourced from Coriell and are reportedly derived from unaffected healthy controls (Table 1).

**Table 1:** Unaffected and FXS affected adult and postnatal human dermal fibroblast cell lines.

ID	Supplier ID	Donor age	Donor gender	Donor ethnicity	Disease status	CGG length
Control 1	GM02673	33	Male	Caucasian	Unaffected	Not reported
Control 2	GM01717	39	Male	Caucasian	Unaffected	Not reported
Control 3	Cell APP 1838	50	Male	Caucasian	Unaffected	Not reported
Control 4	GM01653	37	Male	Caucasian	Unaffected	Not reported
FXS 1	GM05185	26	Male	Caucasian	FXS Affected	800
FXS 2	GM04026	35	Male	Caucasian	FXS Affected	>200
FXS 3	GM09497	28	Male	Caucasian	FXS Affected	>400
FXS 4	GM05131	3	Male	Caucasian	FXS Affected	800,166

**Table 2:** Clinical characteristics of fragile X syndrome donors.

ID	Supplier ID	Phenotypic and behavioral characteristics
FXS 1	GM05185	Not reported
FXS 2	GM04026	Intellectual disability; macro-orchidism
FXS 3	GM09497	Moderate intellectual disability; large ears and long face with prognathic appearance; undefined connective tissue dysplasia
FXS 4	GM05131	Not reported

Phenotypic and behavioral characteristics of fibroblast donors as reported by Coriell Institute for Medical Research.

Pronounced sex-related differences in the cognitive, behavioral, and physical presentation of FXS exist due partially to the influence of random X-chromosome inactivation (XCI) and partial FMRP expression in females (Loesch et al., 2004). Although partial FMRP dosage in heterozygous females is not sufficient to fully restore function, FXS associated phenotypes are typically less severe and less predictable (Tassone et al., 1999). Due to the wide phenotypic variability of FXS presentation in females, the current study utilizes male cell lines to simplify the range of genotype–phenotype associations for the purposes of developing an FXS modelling platform.

Data reported in the following studies include independent biological replicates derived from 3 to 4 individual Control cell lines, and 4 FXS cell lines as reported in the respective figure legends for each study. Due to difficulties with cell expansion following reprogramming, the availability of Control 4 (GM01653) was limited. Sufficient sample was only able to be collected for gene expression analysis at the reprogramming stage and was prioritized for *FMR1*, methylation and FMRP analysis following differentiation. This limited control line availability to *n* = 3 in specific studies as indicated.

**Figure 1:**
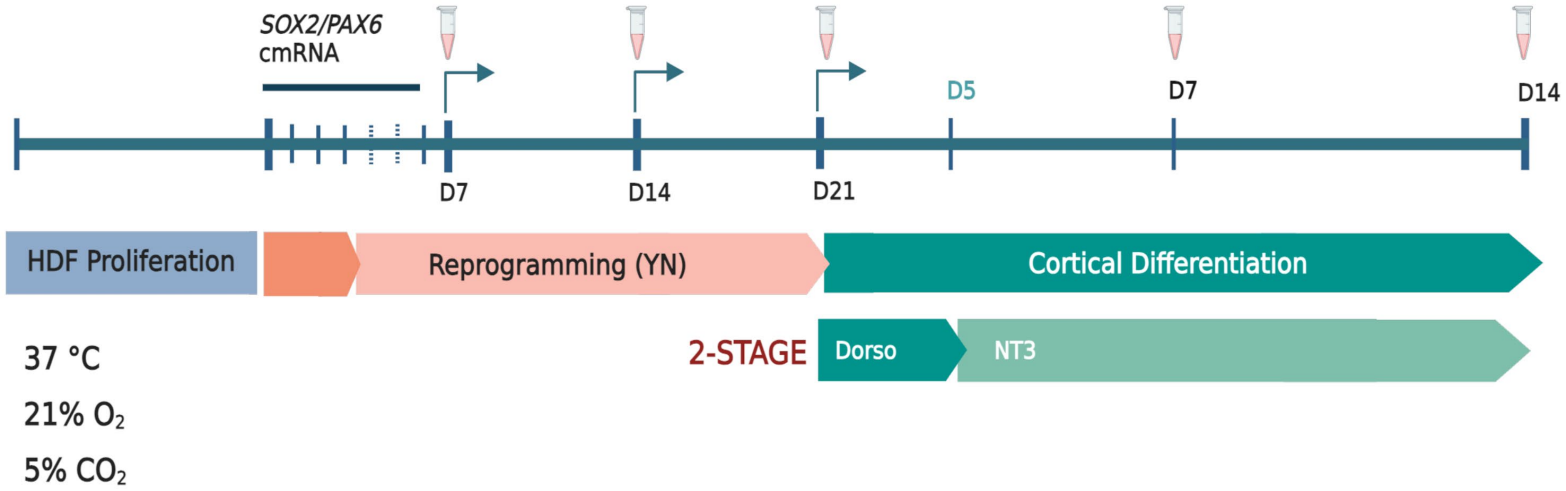
Schematic diagram of *SOX2/PAX6* cmRNA-mediated reprogramming of human dermal fibroblasts (HDFs) to generate human induced dorsal forebrain precursors (hiDFPs) and cortical neurons. Weekly passaging of cells was performed throughout reprogramming only. Samples were collected and/or fixed for analysis at days 7, 14 and 21 of reprogramming. At 21 days of reprogramming cells were seeded onto GelTrex-coated glass coverslips for differentiation and collected and/or fixed for analysis at days 7 and 14. Arrows indicate passaging times. Tube icons indicate sample collection/fixation times. (Y) = Y26732; (*N*) = *N* − 2 supplement. Dorso, dorsomorphin; NT3, neurotropin-3. Schematic created with BioRender.com.

The transient over-expression of the pro-neural genes *SOX2* and *PAX6* using chemically modified mRNA (cmRNA; Ethris GmbH, Munich, Germany) was used to induce a neuronal precursor fate in both adult and postnatal human dermal fibroblasts (HDF; Figure 1). To reduce innate immune response and improve mRNA stability, synthesized *SOX2/PAX6* mRNA was modified through the replacement of uridine and cytidine residues with 2- thiouridine and 5-methylcytidine analogues, respectively (Connor et al., 2018). HDFs were co-transfected with 2.5 μg each of *SOX2* and *PAX6* cmRNA using Lipofectamine™ RNAiMAX (Thermo Fisher Scientific) transfection reagent diluted to 0.02% in Opti-MEM™. Transfection mix (500 μL) was added dropwise to each well to a total volume of 2 mL per well (6-well format) and mixed by gentle manual oscillation of the plate. Transfected cells were incubated at 37°C (21% O_2_, 5% CO_2_) for 5 h, after which time transfection mixture was removed and replenished with 1.5 mL of fresh reprogramming media. Five-hour transfections were conducted every 24 h over four consecutive days. Cells were reprogrammed under normoxic conditions (21% O_2_), 5% CO_2,_ at 37°C in Neurobasal-A media (NBA; Thermo Fisher Scientific) with 1 mM valproic acid (Sigma Aldrich), 1% penicillin- streptomycin (Thermo Fisher Scientific), 1% Glutamax (Thermo Fisher Scientific), 2% B-27 with vitamin-A (Thermo Fisher Scientific), 10 μM retinoic acid (Sigma Aldrich), 2 μg/mL heparin (Sigma Aldrich), 20 ng/mL EGF (ProSpec-Tany TechnoGene), 20 ng/mL FGF2 (ProSpec-Tany TechnoGene), 10 μM Y26732 (Abcam) and 1% N-2 supplement (Thermo Fisher Scientific). A final concentration of 1 μM RepSox (Abcam) was added to the media from day 7 to day 21 of reprogramming. Culture medium was completely replenished every 2 days. Cells were passaged weekly using 0.05% Trypsin–EDTA (Thermo Fisher Scientific) followed by inactivation with trypsin inhibitor (Thermo Fisher Scientific).

Following 21 days of reprogramming, human induced dorsal forebrain precursors (hiDFPs) were disassociated at room temperature with StemPro Accutase™ (Thermo Fisher Scientific) and passaged to GelTrex-coated (Thermo Fisher Scientific) glass coverslips at 60,000–80,000 cells/well (24-well plate format) for differentiation. NBA-based cortical differentiation media contained 1% penicillin–streptomycin, 2% B-27 supplement with retinoic acid, 1% N-2 supplement, 10 μM Y-27632, 10 μM forskolin (Abcam), 20 ng/mL BDNF (Prospec-Tany Technogene), 20 ng/mL GDNF (Prospec-Tany Technogene), and 200 nM ascorbic acid (Sigma-Aldrich). Dorsomorphin, 1 μM (Abcam) was included in the differentiation media from day 0 to day 5 and 10 ng/mL NT3 (Prospec-Tany Technogene) from Day 0 to 14 of differentiation. At days 7 and 14 of differentiation, cells were either collected for gene expression and methylation analysis or were fixed and processed for immunocytochemistry (Figure 1).

### 2.2. Quantitative RT-PCR

Total RNA was extracted from source HDFs before transfection, hiDFPs at 7, 14, and 21 days of reprogramming, and hiDFP-derived neuronal cells at 14 days after cortical differentiation using the Nucleospin RNA kit (Macherey Nagel). Superscript IV reverse transcriptase (Invitrogen) was used to synthesize cDNA from total RNA. Duplex qPCR reactions were performed in triplicate using the TaqMan system (Applied Biosystems) with ribosomal 18S rRNA as the internal standard using an equivalent of 4–10 ng mRNA per reaction. Gene expression was normalized to ribosomal 18S rRNA as the internal standard. Gene expression is presented as either a ΔCT value or a fold change relative to day 0 HDF or day 21 hiDFP. The fold change values were calculated using the comparative Ct (ΔΔCt) method (Livak and Schmittgen, 2001). Briefly, the ΔCt value for a reference sample (18S) was subtracted from the ΔCt value for a sample of interest to give a ΔΔCt value that is converted to fold change (2^−ΔΔCt^) using log2. Where calculated fold change values were below 1, the negative inverse of this value was calculated (−1/fold change) representing downregulation of the gene of interest. Fold change expression above or below a 2-fold change threshold indicated up or downregulated expression relative to either day 0 HDF or day 21 hiDFP. Taqman assays used in this study are indicated in Table 3.

**Table 3:** List of TaqMan assays used for gene expression analysis by RT-qPCR.

Gene	Protein	Description	Assay number
*18S*	18S	Eukaryotic 18S rRNA endogenous control	Hs99999901_s1
*ASCL1*	ASCL1	Achaete-scute family BHLH transcription factor 1	Hs04187546_g1
*POU3F2*	BRN2	Brain-specific homeobox/POU domain protein 2	Hs00271595_s1
*BCL11B*	CTIP2	COUP-TF-interacting protein 2	Hs01102259_m1
*CACNA1C*	CAV1.2	Calcium voltage-gated channel subunit alpha 1C	Hs00167681_m1
*CUX1*	CUX1	Cut like homeobox 1	Hs00738851_m1
*DLG1*	SAP97	Discs large MAGUK scaffold protein 1	Hs00938204_m1
*DLG4*	PSD95	Discs large MAGUK scaffold protein 4	Hs00176354_m1
*DLX2*	DLX2	Distal-less homeobox 2	Hs04194137_s1
*FOXG1*	FOXG1	Forkhead box G1	Hs01850784_s1
*FMR1*	FMRP	FMRP translational regulator 1	Hs00924547_m1
*GAD67*	GAD1	Glutamate decarboxylase 67	Hs01065893_m1
*HCN2*	HCN2	Hyperpolarization activated cyclic nucleotide gated potassium and sodium channel 2	Hs00606903_m1
*MAP2*	MAP2	Microtubule-associated protein 2	Hs00258900_m1
*MAP-T*	TAU	Microtubule associated protein tau	Hs00902194_m1
*NES*	NESTIN	Neuroepithelial stem cell protein	Hs04187831_g1
*NEUROG2*	NGN2	Neural-specific basic helix–loop–helix transcription factor	Hs00702774_s1
*TBR1*	TBR1	T-Box brain transcription factor 1	Hs00232429_m1
*EOMES*	TBR2	T-box brain protein 2	Hs00172872_m1
*TUBB3*	TUJ1	Tubulin beta 3 class III	Hs00801390_s1
*SNAP25*	SNAP25	Synaptosome associated protein 25	Hs00938962_m1
*SYN1*	SYNAPSIN I	Synapsin 1	Hs00199577_m1
*SLC1717*	vGLUT1	Vesicular glutamate transporter 1	Hs00220404_m1

### 2.3. Immunofluorescence

Cells fixed in 4% paraformaldehyde (PFA) at 4°C for 10 min were washed in 1 × phosphate-buffered saline (PBS), and then permeabilized in PBS with 0.5% Triton X-100 for 5 min at room temperature (RT). Solutions used for immunocytochemistry prepared as indicated in Supplementary Figure S1. Cells were blocked with 3% goat serum in PBS containing primary antibodies and incubated overnight at 4°C. Cells were then washed with PBS and incubated with secondary antibodies for 1 h at RT. The following human-specific primary antibodies were used: FOXG1 (anti-rabbit, 1:500; Abcam), Ki67 (anti-rabbit, 1:250; Abcam), NGN2 (anti-mouse, 1:200; R&D Systems), TBR2 (anti-rabbit, 1:200; Abcam), TUJ1 (anti-mouse, 1:500; Biolegend), TUJ1 (anti-rabbit, 1:1,000; Abcam), MAP2 (anti-chicken, 1:2000, Abcam) SYN1 (anti-mouse, 1:250; EMD Millipore), PSD95 (anti-rabbit, 1:250; Synaptic Systems), vGLUT1 (anti-rabbit, 1:250; Synaptic Systems), GABA (anti-rabbit, 1:100; Merck) and S100β (anti-rabbit, 1:250; Abcam). The species-appropriate Alexa Fluor™ secondary conjugated antibodies (1:500; Invitrogen) were used for visualization of the primary antibody. Individual cell nuclei were confirmed using Prolong Diamond antifade mountant containing 4′,6-diamidino-2-phenylindole (DAPI; Thermo Fisher Scientific) as per the manufacturer’s instructions.

All phenotypic markers were imaged on a Nikon TE2000E inverted microscope equipped with a Nikon DS-Ri camera using Nikon NIS-Elements BR 4.50.00 software. A maximum of 10 randomly selected fields of view (FOV; 1,608 × 1,608 pixels) per cell line were captured for cell type quantification. All images captured using the same filter were imaged with the same exposure and gain settings where possible to maintain consistency between samples.

Morphology analysis was performed using the Sholl Analysis algorithm of the FIJI plugin ‘Simple Neurite Tracer’ (SNT; Arshadi et al., 2021). Single channel MAP2 or TUJ1 Images were converted to 8-bit images. Reconstruction of the dendritic arbor in SNT was performed by semi-automatic tracing of neuronal processes between user defined points along a given dendrite using a post-hoc fitting procedure based on fluorescent signal. Dendritic complexity was determined from the number of dendrites intersecting each radial distance from the cell soma at 5 μm intervals.

Synaptic proteins were imaged on a Zeiss LSM 800 Airyscan confocal microscope using a 63x oil immersion lens (numerical aperture [NA] of 1.4) and captured using Zen 2.6 software (Biomedical imaging Resource Unit, University of Auckland). The ImageJ plugin ‘Analyze Particles’ was used to quantify the total number of synaptic puncta. All images were subject to background subtraction, with a rolling ball radius (RBR) of 4. Thresholding was then applied consistently to all images with the same immunolabeling (SYN1, PSD95, vGLUT1) to improve signal to noise ratio. The Watershed algorithm was applied to differentiate multiple puncta in close proximity. Regions of interest (ROI) were selected using a dendritic mask applied to each dendrite using the brush selection function in ImageJ set to a width of 30 pixels. ROI were determined from single channel images of the neuron specific markers MAP2 or TUJ1 and added to the ROI manager for analysis. An overlay of each ROI was then applied to single channel binarized and thresholded images of synaptic puncta to capture all puncta within the ROI determined by respective dendrite markers.

### 2.4. Quantification of FMRP expression

The concentration of FMRP in HDF, hiDFP and differentiated samples was determined using Abcam’s FMRP *in vitro* SimpleStep ELISA^®^ kit according to the manufacturer’s instructions and measured at 450 nm on a BioTek Synergy™ 2 Microplate reader.

### 2.5. Characterization of FMR1 promoter methylation

Genomic DNA (gDNA) was isolated from cultured cells using a NucleoSpin^®^ Tissue Kit (Macherey-Nagel) as per the manufacturer’s instructions. In brief, cell pellets were pre-lysed and homogenized using the lysis buffer and Proteinase K provided (Macherey-Nagel). To bind DNA, lysate was transferred to a NucleoSpin^®^ Tissue column and centrifuged at 1000 *g* for 1 min at room temperature. The silica membrane was washed twice with provided wash buffer and centrifuged at 1000 *g* for 1 min per wash cycle. The silica membrane was dried and residual ethanol removed through an additional centrifuge step at 1000 *g* for 1 min. DNA was eluted from the column using 50 μL of prewarmed buffer BE included in kit (70°C), incubated for 1 min at room temperature and centrifuged for 1 min at 1000 *g.* The elution procedure was modified to enhance the yield and concentration of gDNA by performing two elution steps using half the volume of the 100 μL elution buffer indicated. The concentration and quality of extracted gDNA was determined using a NanoPhotometer^®^ NP80 spectrophotometer.

Characterization of the methylation signature of the human *FMR1* promoter by pyrosequencing was performed by EpigenDX using a novel set of primers ADS1451-FS1 and ADS1451-FS2. The human methylation assay covers 22-CG dinucleotides in the 5-Upstream promoter region of the FMR1 gene (Supplementary Figure S2) based on the Ensembl Gene ID: ENSG00000102081 and the Transcript ID: ENST00000370475. The assay covers a range of −523 to −384 from the transcriptional start site (TSS). Bisulphite conversion, PCR and Pyrosequencing of extracted gDNA were undertaken by EpigenDX as described by the service provider below.

#### 2.5.1. Bisulphite conversion

For DNA methylation analysis, 500 ng of extracted gDNA was bisulfite treated using EZ DNA Methylation Kit (Zymo Research Inc., CA). Bisulfite treated DNA was purified according to the manufacturer’s protocol and eluted to a final volume of 46 μL.

#### 2.5.2. PCR

PCRs were performed using 17 ng of bisulfite treated DNA and 0.2 μM of each primer. One primer was biotin-labeled and HPLC purified in order to purify the final PCR product using sepharose beads.

#### 2.5.3. Pyrosequencing

PCR product was bound to Streptavidin Sepharose HP (GE Healthcare Life Sciences), after which the immobilized PCR products were purified, washed, denatured with a 0.2 μM NaOH solution, and rewashed using the Pyrosequencing Vacuum Prep Tool (Pyrosequencing, Qiagen), as per the manufacturer’s protocol. Next, 0.5 μM of sequencing primer was annealed to the purified single stranded PCR products. 10 μL of the PCR products were sequenced by Pyrosequencing on the PSQ96 HS System (Pyrosequencing, Qiagen) following the manufacturer’s instructions. The methylation status of each CpG site was determined individually as an artificial C/T SNP (Single Nucleotide Polymorphism) using QCpG software (Pyrosequencing, Qiagen). The methylation level at each CpG site was calculated as the percentage of the methylated alleles divided by the sum of all methylated and unmethylated alleles. The mean methylation level was calculated using methylation levels of all measured CpG sites within the targeted region of each gene. Each experiment included non-CpG cytosines as internal controls to detect incomplete bisulfite conversion of the input DNA. In addition, a series of unmethylated and methylated DNA are included as controls in each PCR. Furthermore, PCR bias testing was performed by mixing unmethylated normal DNA with *in vitro* methylated DNA at different ratios (0, 5, 10, 25, 50, 75, and 100%), followed by bisulfite modification, PCR, and Pyrosequencing analysis.

### 2.6. Live-cell calcium imaging

Reprogrammed hiDFP cells were cultured on 24-well, black wall, glass bottom Sensoplates (Greiner Bio-One GmbH) for 14 days prior to live calcium imaging. Cells were loaded with a calcium dye working solution containing NBA medium, 5 μM of Cal-520^®^ AM (Abcam) and 0.04% Pluronic^®^ F-127 (Thermo Fisher Scientific) and incubated at 37°C for 1 h, followed by incubation at room temperature for 30 min. After incubation, the calcium dye working solution was replaced with Hank’s Balanced Salt Solution (HBSS) buffer without phenol red (Thermo Fisher Scientific) which included 1 mM Probenecid (Abcam) and 20 mM HEPES (Thermo Fisher Scientific).

The live cell imaging was conducted at Ex/Em = 490/525 nm, ×10 magnification and exposure set to 600 ms. For each replicate, the calcium fluorescence intensity was recorded for a total of 120 s using a Nikon TE2000E inverted fluorescence microscope equipped with a Nikon Digital Sight DS-Ri2 CMOS sensor color camera using NIS Elements AR (Advanced Research) software. For investigation of the functional properties of FXS hiDFP derived cortical neurons, the cells were stimulated with different concentrations of glutamate (12.5 μM, 25 μM, 37.5 μM, 50 μM).

The live cell calcium imaging analysis was conducted using the Time Series Analyzer V3 plugin in FIJI. Each live cell imaging recording yielded a total of 186 frames which was compressed into increments of 3 slices to have a total of 62 frames per condition. For each no glutamate/glutamate condition, over 100 regions of interest were selected, depending on the number of cells visible within a given field of view. The average intensity values were calculated at each frame. The data was further prepared for analysis by randomly selecting a total of 100 regions of interest for each condition. The average fluorescence intensity was defined as the percentage increase normalized to the baseline fluorescence at 0 s.

### 2.7. Statistical analysis

Statistical analyses were performed using IBM SPSS Statistics version 23 (IBM Corporation, United States). Significance is expressed throughout as follows; * *p* ≤ 0.05; ** *p* ≤ 0.01; *** *p* ≤ 0.001. A *p* > 0.05 is not considered significant. For independent samples t-tests, the *t*-distribution (*t*-test) is expressed as follows; t(*x*) = *y*, where (*x*) refers to degrees of freedom (df) and (*y*) refers to the obtained value of the t-statistic (obtained t-value). For all parametric tests, the distribution of data was first assessed to ensure that data were normally distributed, as assessed by Shapiro- Wilk’s test (*p* > 0.05), and had equal variances as assessed by Levene’s test for homogeneity of variances. All graphs were plotted in GraphPad Prism 7 (GraphPad Prism Software, CA, United States).

## 3. Results

### 3.1. FXS-affected precursors and derived neurons recapitulate characteristic genomic and epigenetic features of FXS

#### 3.1.1. FMR1 promoter expression

Hypermethylation of the *FMR1* promoter leading to impaired *FMR1* gene expression and subsequently loss of FMRP expression are hallmarks of FXS, leading to symptoms characteristic of this disorder. It was therefore important to characterize these parameters in our model system and to ensure expression remained stable throughout reprogramming and differentiation.

**Figure 2:**
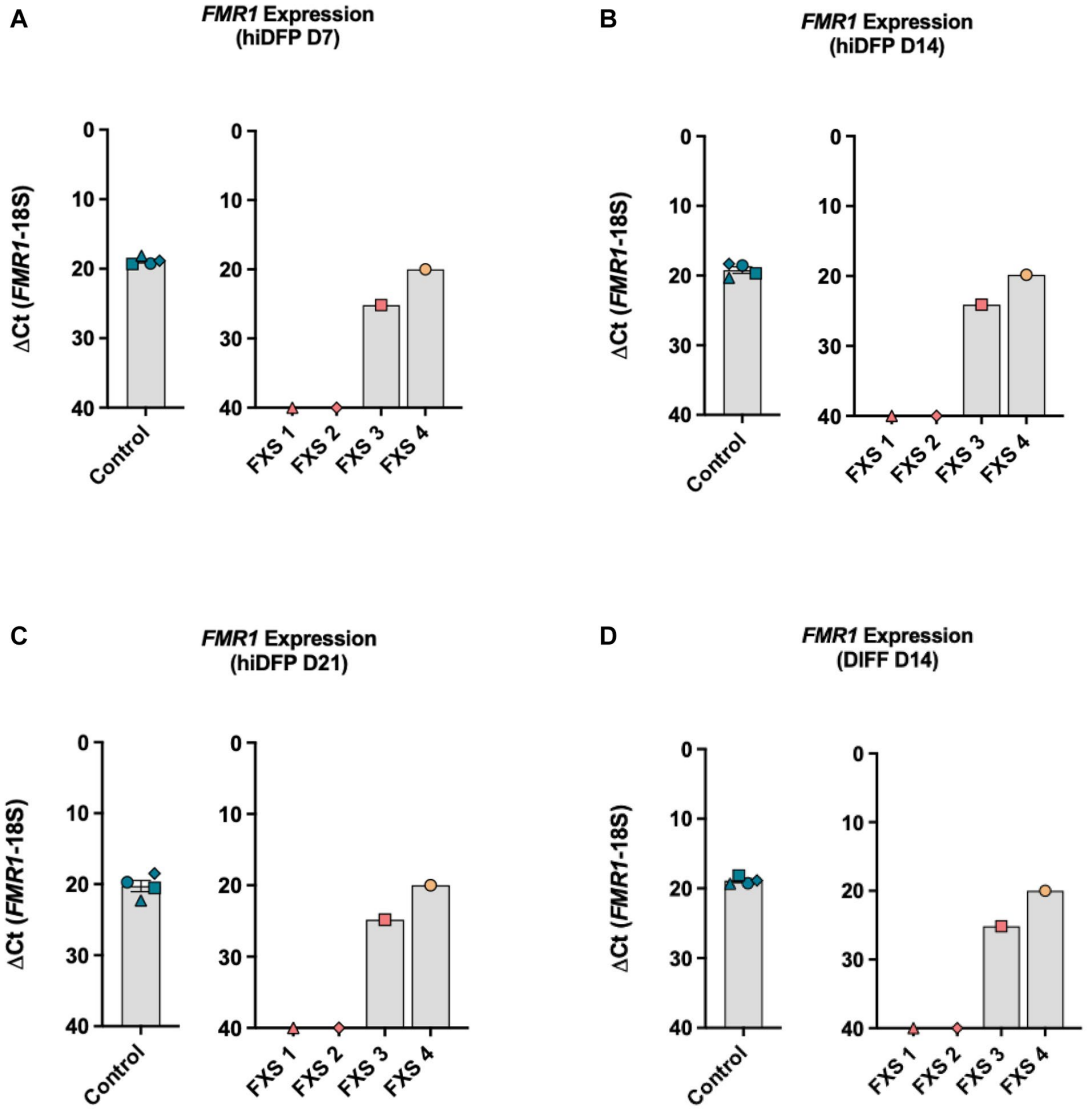
*FMR1* gene expression profile throughout **(A–C)** reprogramming and **(D)** following differentiation. Data are presented as average ∆CT ± SEM for controls, *n* = 4 independent biological replicates. Independent FXS cell lines are plotted individually. The ΔCt value was calculated by subtracting the Ct value for the reference gene (18S) from the Ct value for *FMR1*. Inversion of the y-axis (∆CT 40-0) represents an inverse correlation between the CT value and target abundance with a cutoff threshold of CT 40 representing undetected expression.

The first objective of this study was to confirm the transcriptional status of the *FMR1* promoter in control and FXS-affected cell lines by assessing *FMR1* expression using RT-qPCR. Gene expression analysis by RT-qPCR confirmed the consistent expression of *FMR1* throughout reprogramming in control derived hiDFPs (Figures 2A–C). *FMR1* transcript expression was not detectable by RT-qPCR during reprogramming in two FXS affected cell lines (GM05185 = FXS 1; GM04026 = FXS 2). In contrast, *FMR1* expression was detected in the other two FXS fibroblast cell lines (GM09497 = FXS 3; GM05131 = FXS 4), throughout reprogramming (Figures 2A–C).

Differentiation for 14 days did not alter the *FMR1* expression profile, with abundant and stable transcript expression observed in control lines (Figure 2D). No expression was detected in FXS 1 and FXS 2 but was detected in FXS 3 and FXS 4 at the end of differentiation with levels similar to control lines (Figure 2D). Repeat size and methylation mosaicism can result in *FMR1* expression in FXS affected individuals. To determine whether the expression of *FMR1* detected in FXS 3 and FXS 4 was consistent with mosaicism, methylation analysis of the promoter was undertaken in the following study.

#### 3.1.2. FMR1 promoter methylation signature

*FMR1* expression is regulated by methylation of the CpG enriched gene promoter in FXS. CpG islands are linear sequences of DNA characterized by a high ratio of Cytosine Guanine dinucleotide residues (GC:CG). Methylation of these CpG dinucleotide enriched regions of the promoter is typically associated with transcriptional repression and represents important gene regulatory elements in the mammalian genome that are also implicated in disease associated epigenetic regulation. A molecular hallmark of full (CGG) mutation FXS carriers is hypermethylation of the CpG island located in the 5′ upstream promoter region of the *FMR1* gene and the expanded (CGG) repeat tract. Given methylation of this region correlates with the stability of the CGG repeat mutation as well as phenotype severity, it is crucial to characterize the methylation signature of the *FMR1* promoter throughout development in all cell lines used for modelling FXS.

To confirm the presence of an FXS-associated epigenotype and to determine the impact of direct reprogramming on the maintenance of this methylation signature, bisulfite pyrosequencing was used to characterize the CpG-rich promoter of the *FMR1* gene in HDFs, directly reprogrammed neural precursors and neurons.

In all subsequent studies, FXS cell lines were grouped according to their promoter methylation characteristics in the following manner. FXS- denotes cell lines with an *FMR1* promoter exhibiting an average % methylation of ≥70% (hypermethylated). FXS+ denotes the individual FXS cell line exhibiting <60% promoter methylation.

**Figure 3:**
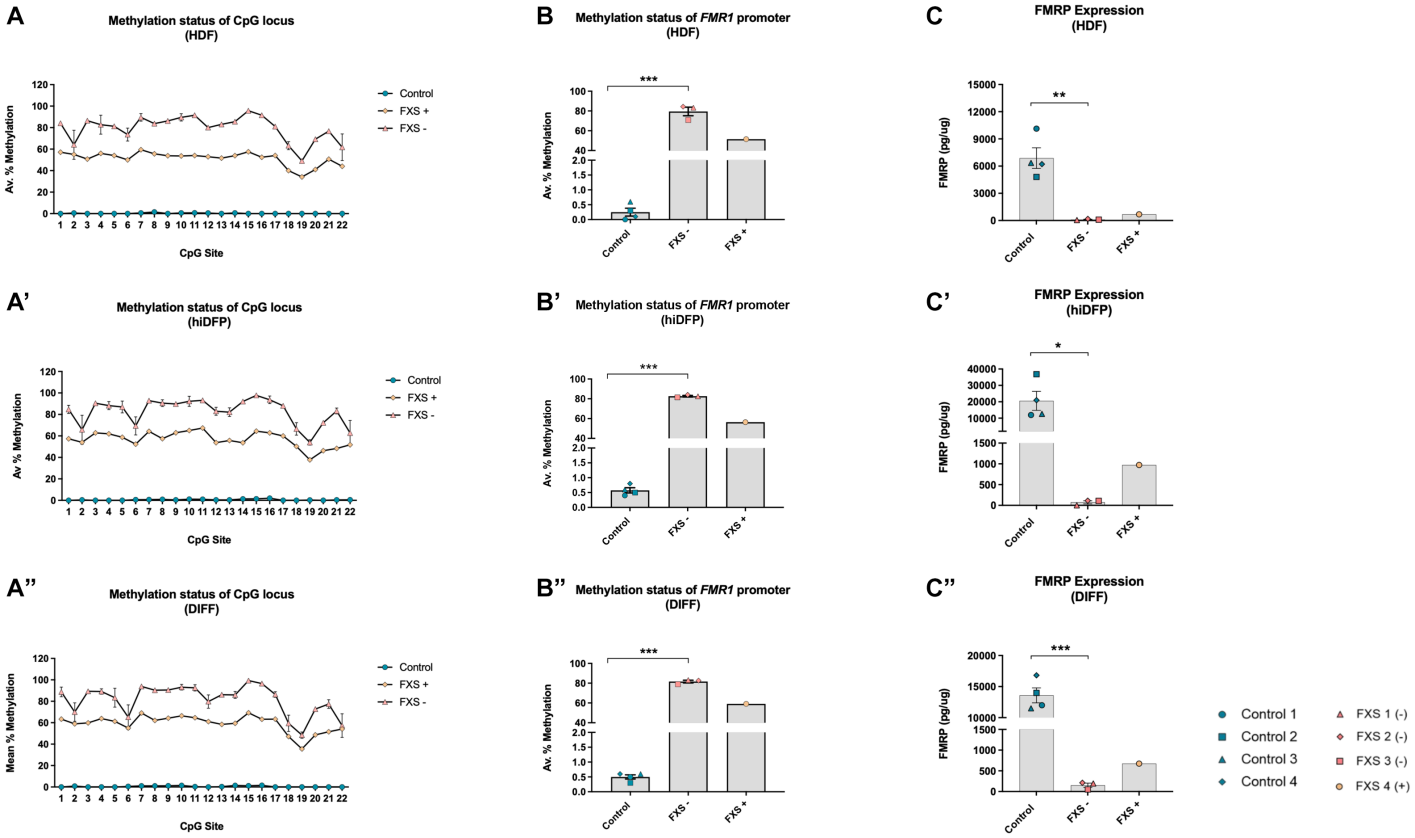
The methylation signature of the 5’upstream *FMR1* promoter is maintained throughout *SOX2/PAX6* cmRNA-mediated reprogramming and cortical differentiation **(A,A’,A”)**. Data depicts the average percent methylation at each of the 22-CpG sites in the *FMR1* promoter assayed at the **(A)** HDF, **(A’)** neural precursor, and **(A”)** neuron stage of development. **(B,B’,B”)** Data represents the average percent methylation across all 22-CpG sites in the *FMR1* promoter at the (B) HDF, **(B’)** neural precursor and **(B”)** neuron stage. FMRP expression profile for **(C)** HDFs, **(C’)** day 21 neural precursors, and **(C”)** differentiated neurons. Data are presented as total FMRP (pg/μg) protein ± SEM. FXS lines were grouped to account for the unique mosaic characteristics of FXS 4, resulting in Control (*n* = 4); FXS- (*n* = 3); FXS+ (*n* = 1) biological replicates from independent cell lines. Statistical significance was determined by an independent samples *t*-test. * *p* < 0.05, ** *p* < 0.01, *** *p* < 0.001. FXS- denotes FXS affected cell lines with an average % methylation of the *FMR1* promoter ≥70%. FXS+ denotes the FXS line exhibiting <60% promoter methylation. FXS 4 was not included in statistical analysis. All data presented as mean ± SEM.

Source fibroblasts from control cell lines exhibited a methylation range from 0.0–3.1% across all 22 CpG sites of the *FMR1* promoter (Figure 3A), with an average of 0.25% ± 0.12% (*n* = 4) prior to reprograming (Figure 3B). FXS- fibroblasts (HDFs) exhibited a methylation range from 49.0–95.7% (Figure 3A), with an average of 80.0% ± 4.33% (*n* = 3; Figure 3B). Fibroblasts from the FXS+ cell line exhibited a methylation range between 34.2–59.4% (Figure 3A), and 51.5% promoter methylation averaged across all 22 CpG sites (*n* = 1; Figure 3B).

Following 21 days of reprogramming, control neural precursors (hiDFPs) exhibited a methylation range from 0 to 2.5% (*n* = 4; Figure 3A’). In contrast, the methylation range for FXS- hiDFPs was between 53.9–97.8% (Figure 3A’), with an average of 82.7% ± 0.77% (*n* = 3; Figure 3B’). FXS+ hiDFPs exhibited a methylation range from 37.8 to 67.4% (Figure 3A’), with an average of 56.4% methylation across all 22 CpG sites (*n* = 1; Figure 3B’).

Following the differentiation of hiDFPs to cortical neurons, differentiated controls exhibited a methylation range from 0–2.6% (Figure 3A”), with an average of 0.5% ± 0.08% (*n* = 4) methylation (Figure 3B”). FXS- hiDFP derived neurons exhibited a methylation range from 48.5 to 99.4% (Figure 3A”), with an average of 81.6% ± 1.31% (*n* = 3; Figure 3B”). FXS+ hiDFP derived neurons exhibited a methylation range from 35.6 to 69.3% (Figure 3A”), with an average of 59.1% methylation across all 22 CpG sites (*n* = 1; Figure 3B”).

Interestingly, despite the expression of *FMR1* in FXS 3, the methylation profile of this cell line closely approximates the CpG methylation signature of the FXS- cohort, with an average percent methylation of 70.90% ± 2.41% (HDFs), 81.5% ± 2.5% (hiDFPs), and 79.0% ± 3.11% in hiDFP-derived neurons. Based on the methylation profile reported in this study (Figures 3A,B”), and the known full CGG repeat mutation size reported previously (Table 1), FXS 3 does not exhibit characteristic traits of mosaicism. In contrast, FXS 4 exhibited a methylation profile (Figures 3A,B”) and repeat size (Table 1) consistent with mosaicism. For this reason, FXS 4 (FXS+) was analyzed independently from the FXS- cohort.

An independent samples T-test indicated a significant difference in promoter methylation between control and FXS- HDFs, with FXS lines exhibiting a higher average percentage methylation than controls prior to reprogramming [*t*(3.00) = −9.45, *p* = 0.003; Figure 3B]. Notably, a significant difference in the methylation of the *FMR1* promoter in the FXS- cohort compared to control hiDFPs was preserved following reprogramming, with FXS- lines exhibiting higher average methylation than control lines [*t*(3.00) = −11.45, *p* = 0.001; Figure 3B’]. A significant difference in the average promoter methylation was also detected between control and FXS lines following differentiation [*t*(3.00) = −13.220, *p* = 0.001; Figure 3B”].

Together these results indicate that the FXS associated status of source fibroblasts is maintained throughout reprogramming and differentiation, with FXS affected neural precursors and derived neurons shown to recapitulate the epigenetic signatures characteristic of the FXS affected *FMR1* promoter. Additionally, this epigenetic signature is preserved in the partially methylated mosaic cell line (FXS 4) irrespective of developmental stage.

#### 3.1.3. FMRP expression

While the loss of a transcriptionally active *FMR1* promoter due to hypermethylation is a hallmark of FXS, the phenotypic effects of *FMR1* silencing are mediated by the loss or aberrant expression of the protein FMRP. To determine whether FXS affected cell lines recapitulate and maintain an FMRP expression profile characteristic of FXS, total protein expression was determined prior to, and following reprogramming and differentiation.

A significant difference in total FMRP between control and FXS HDFs was detected, with FXS- lines exhibiting a lower total amount of FMRP compared to control lines prior to reprogramming, [*t*(7.00) = 5.248, *p* ≤ 0.001; Figure 3C], and following reprogramming [*t*(4.00) = 3.091, *p* = 0.030; Figure 3C’]. Following 14 days of differentiation, FXS- hiDFP derived neurons also showed a significant decrease in total FMRP compared to control lines [*t*(5.00) = 9.407, *p* = 0.001; Figure 3C”]. While FXS 4 exhibited elevated *FMR1* expression and reduced promoter methylation, this line expressed reduced total FMRP levels comparable to the FXS- cohort, features that are consistent with mosaicism.

Interestingly, limited examples of residual *FMR1* transcript expression have been reported in non-mosaic, full mutation FXS affected cell lines (Tassone et al., 2001). The mechanism and downstream impact of residual gene expression is unclear but is linked to inefficient translation of FMRP owing to the expanded repeat tract in the mutant transcripts (Tassone et al., 2001; Ludwig et al., 2014). Consistent with this observation, FMRP expression in the FXS 3 cell line was impaired throughout reprogramming and differentiation despite the presence of *FMR1* expression. Given the characteristic phenotypes of FXS are mediated by the loss of FMRP and its downstream regulatory functions, protein expression was an important consideration for the inclusion of the FXS 3 cell line in the FXS- cohort in subsequent analysis. However, further analysis of the possible phenotypic impact of residual mutant *FMR1* transcript would be informative.

### 3.2. *SOX2/PAX6* cmRNA direct reprogramming enables the generation of FXS-affected dorsal forebrain precursor cells

#### 3.2.1. Morphological properties

**Figure 4:**
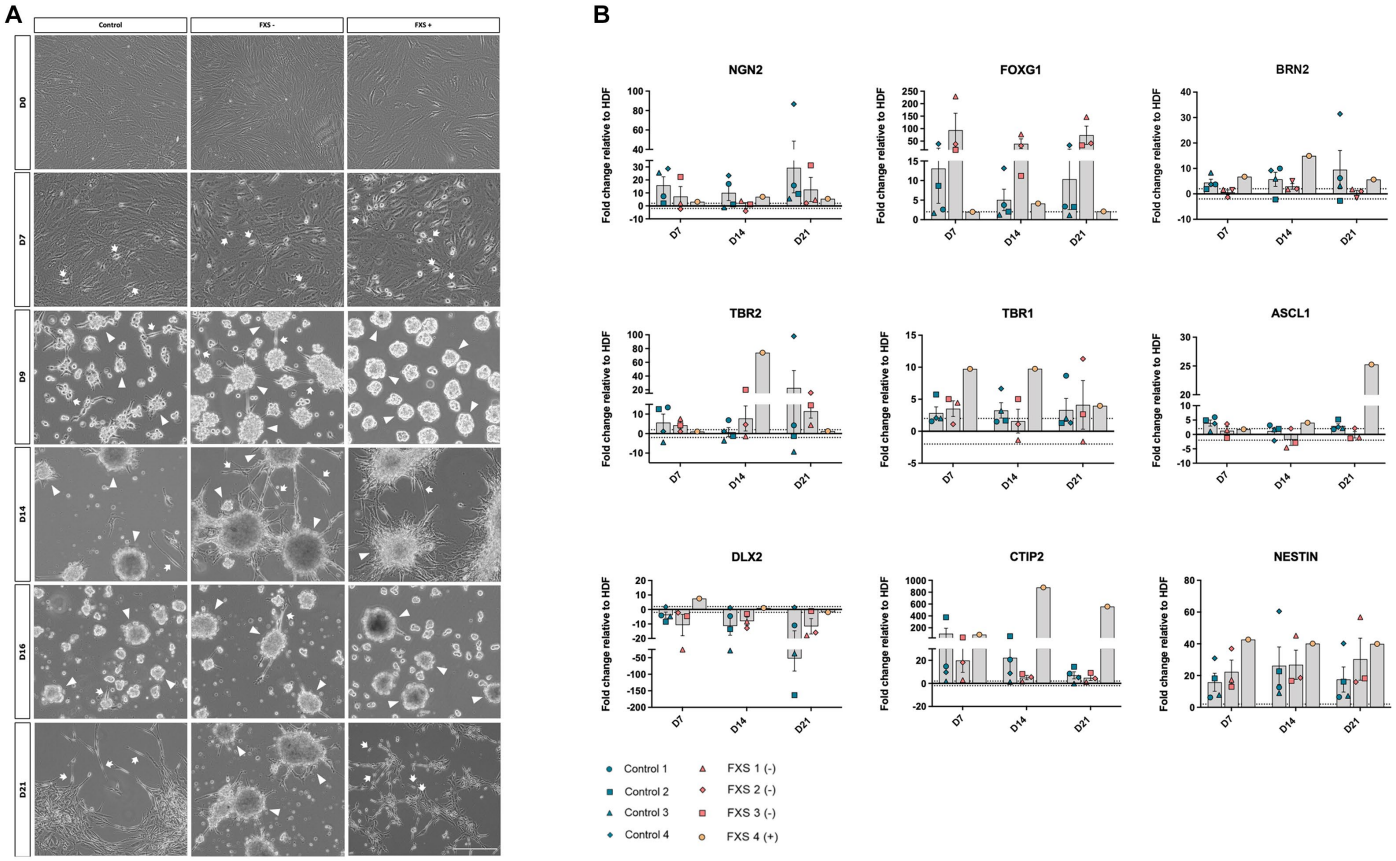
**(A)** Phase contrast images depicting morphological characteristics of hiDFPs throughout reprogramming. Representative images from one cell line per cohort are depicted (Control GM02673; FXS- GM05185; FXS+ GM05131). Images represent cell morphology immediately prior to weekly passaging and 2-days post-passage. Arrow heads indicate examples of cell aggregates; arrows indicate polarized cells. Scale = 200 μm. **(B)** Temporal gene expression profile throughout reprogramming. Data are presented as mean fold change relative to HDF ± SEM, Control (*n* = 4); FXS- (*n* = 3); FXS+ (*n* = 1) biological replicates from independent cell lines. Dotted line indicates ±2-fold threshold for fold change expression.

To confirm the ability of *SOX2/PAX6* direct reprogramming to generate FXS-affected dorsal forebrain precursors we examined the morphological characteristics of both control and FXS human cell lines throughout reprogramming. Prior to transfection, HDFs across all cell lines exhibited large, flat, and elongated morphologies typical of fibroblasts (Figure 4A). Throughout reprogramming morphological changes were observed in both control and FXS affected cells. Prior to passaging at day 7 of reprograming both control and FXS neural precursors exhibited a flattened spindle-shaped fibroblast-like morphology alongside intermittent bipolar precursor-like like cells (Figure 4A). Over time in culture cells progressively acquired neural precursor-like morphologies including individual bipolar cells, discrete aggregates of semi-adherent or free floating neurosphere- like colonies. Prior to passaging at day 14 of reprogramming (Figure 4A), free floating neurospheres had acquired more adherent properties, with bipolar precursors migrating outwards in both control and FXS-derived cultures. Prior to plating for differentiation at 21 days of reprogramming (Figure 4A), free floating neurospheres in both control and FXS+ neural precursors had fully adhered and dispersed and were characterized by an abundance of multipolar precursor-like cells. While FXS+ neural precursors exhibited characteristics comparable to controls, FXS- neural precursors maintained semi-adherent neurosphere-like characteristics throughout reprogramming.

#### 3.2.2. Gene expression profile

To confirm the ability of *SOX2/PAX6* cmRNA direct reprogramming to promote specification to a dorsal forebrain precursor fate, we examined the temporal profile of gene expression signatures characteristic of dorsal forebrain precursor development (Molyneaux et al., 2007; Figure 4B). While the average expression of the pro-neural transcription factor, *FOXG1* was upregulated in both control and FXS-affected precursors, FXS- cell lines exhibited robust early upregulation at day 7, which remained highly elevated throughout reprogramming (Figure 4B). The average expression of *NGN2* and *BRN2* was upregulated over time in control neural precursors. In contrast, the average expression of *NGN2* did not increase over time in *FMR1* deficient FXS- cell lines with a modest, transient upregulation of *BRN2* expression detected in FXS- affected precursors at day 14 of reprogramming only (Figure 4B). Low expression of the dorsal forebrain transcription factor, *TBR2*, was detected in control derived neural precursors from days 7 through day 14 of reprogramming with an increase in *TBR2* expression only observed at day 21 of reprogramming (Figure 4B). *TBR2* expression was similarly low in FXS- affected precursors at day 7 and day 14 of reprogramming, before increasing slightly at day 21 of reprogramming (Figure 4B). In contrast, FXS+ affected precursors expressed upregulation of TBR2 at day 14 before expression decreased by day 21 (Figure 4B). In control derived precursors, low average expression of the transcription factor *TBR1* was detected early in reprogramming as expected (Figure 4B). However, *TBR1* expression remained consistently low in control cell lines, with little change detected between day 7 and day 21 of reprogramming. Similarly, a modest upregulation of *TBR1* expression was detected in FXS- affected precursors at day 7, decreasing below the ±2-fold change expression threshold relative to HDFs at day 14 (Figure 4B). Although the average expression of *TBR1* increased over time in the FXS- cohort, expression was highly variable between cell lines. In contrast, early upregulation of *TBR1* was detected in FXS+ affected precursors before decreasing to expression levels comparable to control line by the end of reprogramming (Figure 4B).

The pro-neural transcription factor *ASCL1*, has been implicated in the specification of ventral telencephalic identity by regulating the expression of both *DLX* and *GAD* genes. *ASCL1* is also thought to identify a population of dorsal precursors that give rise to glutamatergic neurons. It was surprising therefore, that *ASCL1* expression remained low in control precursors and unchanged in FXS- affected precursors by 21 days of reprogramming (Figure 4B). Expression of the ventral telencephalic transcription factor *DLX2* was downregulated or otherwise exhibited no expression change relative to HDF in both control and FXS affected precursors throughout reprogramming (Figure 4B). Upregulated expression of the multi-lineage transcription factor *CTIP2* was detected in control derived precursors, with decreasing expression observed from day 7 to day 21 of reprogramming (Figure 4B). While *CTIP2* expression was upregulated relative to HDFs in FXS- affected precursors, expression remained only marginally above the 2-fold threshold for expression. In contrast, high levels of *CTIP2* expression was observed in FXS+ derived precursors throughout reprogramming (Figure 4B). Expression of the transcription factor *MEIS2*, a marker of ventral precursor development, was not upregulated relative to HDFs over time in either control or FXS neural precursors, remaining within the 2-fold expression threshold throughout, reinforcing the acquisition of a dorsal rather than ventral precursor fate (data not shown). Finally, while considerable variability in the expression of the generic neural precursor gene *NESTIN* was detected between replicates within both control and FXS cohorts, highly elevated expression was detected throughout reprogramming in both control and FXS affected precursors (Figure 4B).

In summary, the upregulation of the key transcription factors *NGN2, BRN2, TBR2* and *TBR1* involved in regulating dorsal forebrain specific neural precursor development coupled with downregulation of markers of ventral forebrain specification were detected throughout reprogramming.

#### 3.2.3. Immunocytochemistry

**Figure 5:**
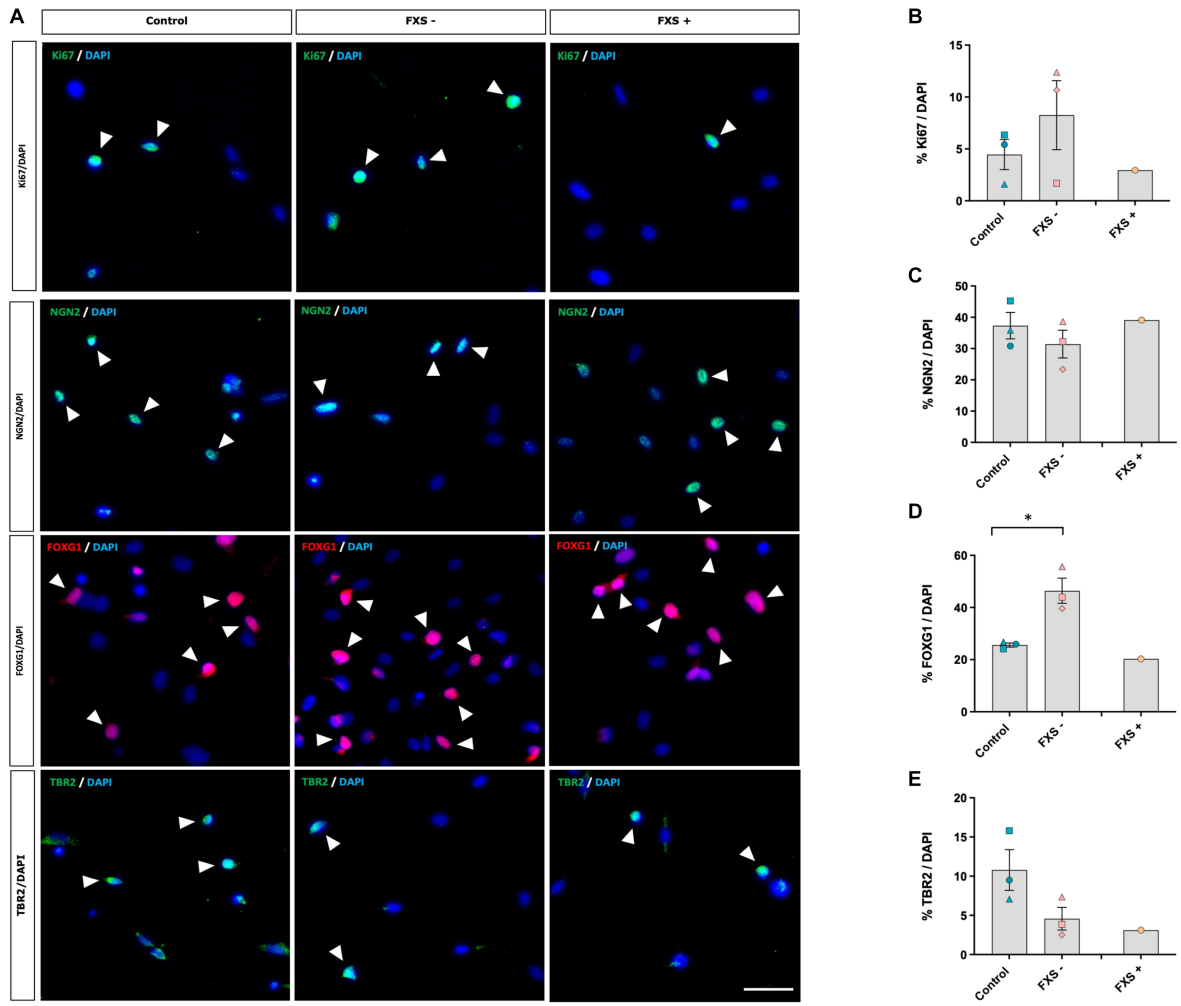
*SOX2/PAX6* cmRNA-derived neural precursors express phenotypic markers characteristic of dorsal forebrain precursor development. **(A)** Expression of the proliferative marker Ki67 and cortical precursor proteins NGN2, FOXG1 and TBR2. Arrow heads indicate examples of immuno-positive cells. Scale bar = 50 μm. **(B–E)** Quantification of protein markers. Data are presented as mean ± SEM, control (*n* = 3), FXS- (*n* = 3), FXS+ (*n* = 1) biological replicates from independent cell lines. Statistical significance was determined by an independent samples *t*-test * *p* < 0.05. FXS+ not included in statistical analysis.

Immunocytochemistry was used to confirm whether *SOX2/PAX6* cmRNA-derived neural precursors express phenotypic markers indicative of dorsal forebrain precursor development (Figure 5A). The proportion of neural precursors immuno-positive for cell cycle and lineage-specific markers was quantified relative to the number of DAPI+ cells in both control and FXS-affected neural precursors to determine whether significant FXS-associated differences in proliferation and regional specification were present in our model.

Following 21 days of reprogramming no difference in the expression of the proliferative marker, Ki67, was observed between FXS- neural precursors and control precursors (Figure 5B). Expression of the stem cell markers NANOG, TRA-160, and SSEA were not detected by immunocytochemistry in either control or FXS-affected neural precursors (Supplementary Figure S3), indicating that control and FXS-affected neural precursors did not transit through a pluripotency stage prior to differentiation.

Immunocytochemistry confirmed a telencephalic dorsal forebrain regional identity in both control and FXS neural precursors after 21 days in reprogramming (Figure 5A). No significant difference in the expression of the forebrain specific transcription factor NGN2 was observed between control and FXS- neural precursors (Figure 5C). FOXG1 is a transcription factor involved in early forebrain patterning and notably implicated in the progression of autism spectrum disorder development. Interestingly, a significant increase in FOXG1 expression was detected in FXS- neural precursors compared to control [*t*(4) = −3.57, *p* = 0.023; Figure 5D]. Independent confirmation by Western blot would provide a useful validation of differential protein expression in follow up analysis. TBR2, an important transcription factor involved in cortical neurogenesis that is enriched in intermediate neural precursors of the dorsal forebrain, was expressed in both control and FXS- neural precursors (Figure 5E). No significant difference in the expression of TBR2 was observed between control and FXS- neural precursors after 21 days of reprogramming.

These results confirm the capability of our unique *SOX2/PAX6* cmRNA-based reprograming approach to generate both control and FXS-affected human induced dorsal forebrain precursors (hiDFPs).

### 3.3. Characterizing the gene expression profile of control and FXS hiDFP-derived neurons following differentiation

**Figure 6:**
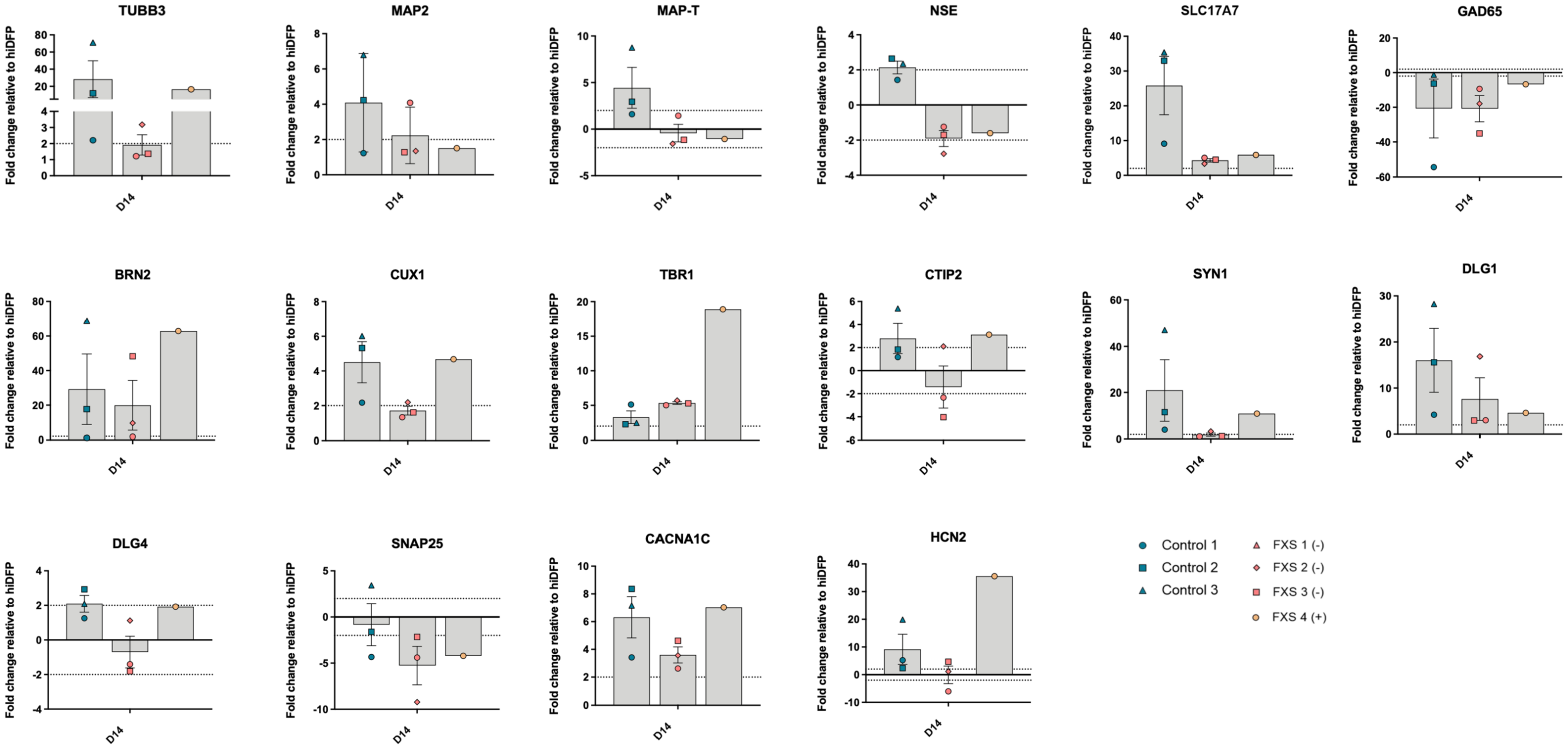
Gene expression profile of hiDFP-derived cortical neurons following 14 days of differentiation. Data are presented as mean fold change relative to day 21 of reprogramming (hiDFP D21) ± SEM, Control (*n* = 3); FXS- (*n* = 3); FXS+ (*n* = 1) biological replicates from independent cell lines. Dotted line indicates a ± 2-fold threshold for fold change expression.

The ability of our cortical differentiation protocol to promote the upregulation of genes required for subtype specific neuron specification and synaptic maturation in both control and FXS hiDFP-derived neurons was evaluated by RT-qPCR (Figure 6). Following 14 days of differentiation, upregulation of the immature neuronal gene, *TUBB3,* and the mature neuronal gene, *MAP2,* relative to day 21 hiDFP, indicated the commitment of control and FXS hiDFPs towards a neuronal lineage (Figure 6). Interestingly, FXS+ hiDFP-derived neurons exhibited a 16.62-fold upregulation of *TUBB3*, but no change in expression of MAP2 relative to day 21 hiDFPs. Control and FXS hiDFP-derived neurons exhibit ΔCT values indicative of moderate transcript abundance for both *TUBB3* (Control ΔCT 17.42; FXS ΔCT 18) and *MAP2* (Control ΔCT 23.38; FXS- ΔCT 23.46). However, in FXS-derived neurons the relative fold change expression of *TUBB3* (1.92 ± 0.63) does not exceed the ±2-fold change threshold for gene expression. Similarly, the fold-change expression of *MAP2* (2.23 ± 0.93) only marginally increased above this expression threshold in FXS- cells. This may indicate altered progression of FXS hiDFPs towards a neuronal lineage compared to controls This is consistent with the inability of FXS hiDFP-derived neurons to express genes encoding either neuron-specific enolase (*NSE*) or the microtubule associated protein Tau (*MAP-T*) above a 2-fold expression threshold (Figure 6). Similarly, FXS+ hiDFP-derived neurons did not demonstrate a change in either *NSE* or *MAP-T* expression relative to hiDFPs above the threshold. In contrast, control hiDFP-derived neurons demonstrated an increased fold change expression of both *NSE* and *MAP-T* expression.

Upregulation of the *SLC17A7* gene encoding vGLUT1 protein in control and FXS- hiDFP-derived neurons relative to day 21 hiDFPs is indicative of hiDFP progression towards a glutamatergic lineage following 14 days of differentiation. This is corroborated by downregulation of glutamate decarboxylase (*GAD65*) in control and FXS hiDFP-derived neurons. Progression of dorsal forebrain precursors along a cortical neuron trajectory is consistent with upregulated gene expression of *BRN2* (layer II) and *CUX1* (layer III), corresponding with late-stage upper layer neuron development (McEvilly et al., 2002; Sugitani et al., 2002; Bulchand et al., 2003), as well as *TBR1* (layer VI) and *CTIP2* (V), indicating the presence of deep layer neurons (Hevner et al., 2001; Molyneaux et al., 2007). Both control and FXS hiDFP-derived neurons expressed upregulation of *BRN2* following 14 days of differentiation. Expression of the upper layer neuronal gene *CUX1* was also upregulated in control hiDFP-derived neurons but did not change relative to hiDFPs in FXS- hiDFP-derived neurons. Interestingly, FXS+ hiDFP-derived neurons exhibited a *CUX1* expression level comparable to controls. In addition to the presence of upper layer neuronal gene signatures, control hiDFP-derived neurons also exhibited upregulated expression of *TBR1* and *CTIP2*, both of which are involved in the specification of deep layer neurons (Hevner et al., 2001; Molyneaux et al., 2007). *TBR1* was upregulated in both control and FXS hiDFP-derived neurons, with robust upregulation in FXS+ hiDFP-derived neurons. In contrast, while *CTIP2* was upregulated in control hiDFP-derived neurons, FXS- cell lines exhibited no change in *CTIP2* expression while FXS+ hiDFP-derived neurons expressed an increase in *CTIP2* relative to hiDFPs.

The expression of synapse-related and ion-channel specific genes required for functional maturation was also investigated following 14 days of differentiation (Figure 6). Control hiDFP-derived neurons exhibited increased expression of the *SYN1* gene, which encodes a presynaptic neuronal phosphoprotein involved in vesicular trafficking and neurotransmitter release. In contrast, no change in *SYN1* expression was detected in FXS- hiDFP-derived neurons relative to day 21 hiDFPs, despite transcript expression comparable to controls (control, ΔCT 20.07; FXS-, ΔCT 21.08). In contrast, increased expression of *SYN1* was detected in FXS+ hiDFP-derived neurons compared to hiDFPs. Elevated expression of the *DLG1* gene was detected in control hiDFP-derived neurons and FXS hiDFP-derived neurons. *DLG1* encodes the scaffolding protein, synapse associated protein-97 (SAP97). Expression of the related synaptic gene *DLG4* was also examined. *DLG4* encodes the postsynaptic scaffolding protein PSD95 involved in the assembly of signaling complexes in excitatory neurons. *DLG4* expression was increased in control and FXS+ hiDFP-derived neurons, but not in FXS- hiDFP-derived neurons. No change in the expression of *SNAP25*, a gene encoding an important synaptic protein regulating neurotransmitter release in both glutamatergic and GABAergic neurons was detected in control derived neurons. In contrast, reduced expression of *SNAP25* was detected in both FXS- and FXS+ hiDFP-derived neurons. Expression of the L-type voltage-gated calcium channel, *CACNA1C* was elevated in both control and FXS hiDFP-derived neurons. An increase in the expression of the voltage-gated cation channel gene, *HCN2* was detected in control hiDFP-derived neurons. However, increased *HCN2* gene expression was not detected in FXS- hiDFP-derived neurons after 14 days of differentiation. Interestingly, enhanced expression of *HCN2* was detected in FXS+ hiDFP-derived neurons with a 35.55-fold increase in expression compared to hiDFPs.

These findings indicate that our cortical differentiation protocol of hiDFPs generates a population of cortical neurons exhibiting diverse layer specific identities and expressing appropriate synaptic and ion channel gene markers. Interestingly, gene expression for many of these markers was lower or exhibited no change relative to hiDFP in FXS- neurons. In contrast, FXS+ neurons expressed a comparable or higher expression level than control neurons. This suggests an impairment of neurogenesis in FXS- hiDFP-derived neurons.

### 3.4. Phenotypic characterization of FXS hiDFP-derived cells following differentiation

**Figure 7:**
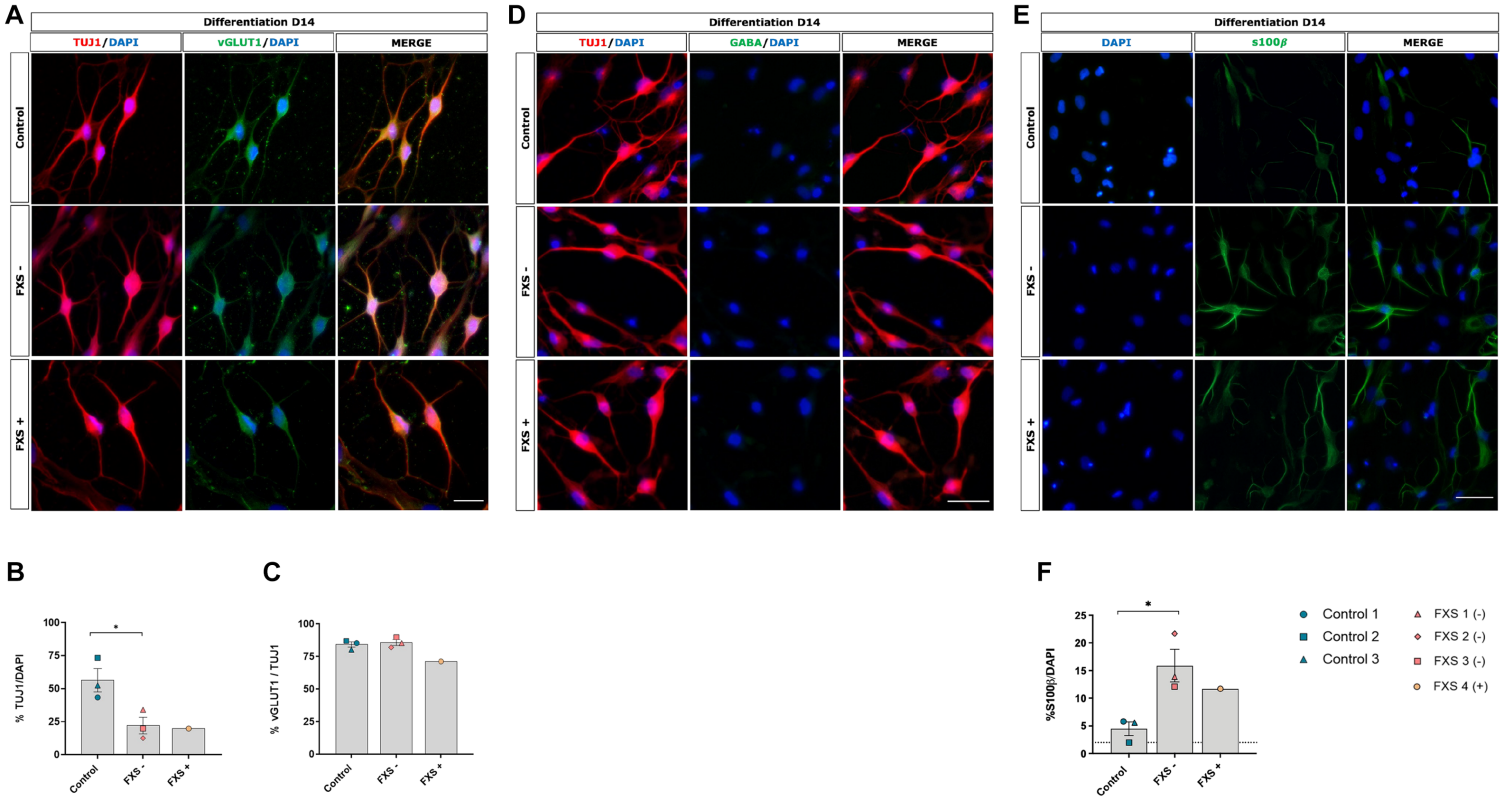
Phenotypic characterization of control and FXS hiDFP-derived neurons following 14 days of differentiation **(A,D,E)**. Scale bar = 100 μm **(A,D)**; 50 μm **(E)**. **(B,C,F)** Quantification of TUJ1 **(B)**, vGLUT1 **(C)** and s100β **(F)** expression in control and FXS hiDFP-derived neurons as a percentage of DAPI+ cells. Data presented as the average percentage of immuno- positive cells ± SEM, control (*n* = 3); FXS- (*n* = 3) biological replicates from independent cell lines Statistical significance was determined by an independent samples *t*-test, * *p* < 0.05. FXS+ (*n* = 1) not included in statistical analysis.

Neuronal specification and maturation are known to be dysregulated in FXS (Castren et al., 2005; Luo et al., 2010; Guo et al., 2011; Sunamura et al., 2018; Brighi et al., 2021). To confirm the ability of our method to generate glutamatergic cortical neurons, and to assess any FXS associated changes in fate specification, immunocytochemistry was used to confirm the phenotypic lineage of both control and FXS hiDFP-derived neurons. Robust expression of the immature neuronal marker TUJ1 was detected by 14 days of differentiation (Figure 7A). Following 14 days of differentiation, the neuronal yield determined for FXS- hiDFP-derived TUJ1+ neurons was significantly less than that for control hiDFP-derived neurons [*t*(4) = 3.163, *p* = 0.034; Figure 7B]. Similarly, a relatively low neuronal yield of 19.9% (*n* = 1) was detected following the differentiation of FXS+ hiDFP-derived TUJ1+ neurons.

To confirm that hiDFP- derived neurons acquired a glutamatergic phenotype and assess whether an FXS-associated bias in neuronal subtype specification existed, the proportion of TUJ1+ cells co-expressing the vesicular glutamate transporter vGLUT1 was determined. Both control and FXS hiDFP-derived neurons exhibited colocalized expression of vGLUT1 with TUJ1 (Figure 7A), with 84.12% ± 1.68% of TUJ1+ control neurons co-expressing vGLUT1 and 86.64% ± 2.54% of FXS lines exhibiting vGLUT1+/TUJ1+ neurons (Figure 7C). No significant difference in the percentage of vGLUT1+/TUJ1+ neurons was determined between control and FXS- hiDFP-derived neurons at day 14 of differentiation (Figure 7C). Similarly, a high proportion (80.58% ± 2.69%) of FXS+ hiDFP-derived TUJ1+ neurons were glutamatergic after 14 days of differentiation (Figure 7C). In contrast, GABA expression was not detected in either control or FXS hiDFP-derived neurons (Figure 7D). This is consistent with the transcriptional profile of differentiated neurons, indicating robust downregulation of the catalytic enzyme, *GAD65* (Figure 6) required for GABA production during gene expression analysis. Expression of S100β, a marker of non-reactive astrocytes, was detected in both control and FXS hiDFP-derived neuronal cultures (Figure 7E). Interestingly, the percentage of S100β+ / DAPI cells was significantly higher in FXS- hiDFP-derived neurons (15.88% ± 1.61%) compared to controls (4.46% ± 0.57%) at day 14 of differentiation [*t*(4) = 3.587, *p* = 0.023; Figure 7F].

Overall, phenotypic analysis indicates that while FXS hiDFP-derived neurons do not exhibit an altered developmental trajectory towards a glutamatergic lineage compared to controls, they do exhibit an increased percentage of non-reactive astrocytes with a corresponding reduction in neurons.

### 3.5. Investigation of the functional properties of FXS hiDFP-derived cortical neurons

**Figure 8:**
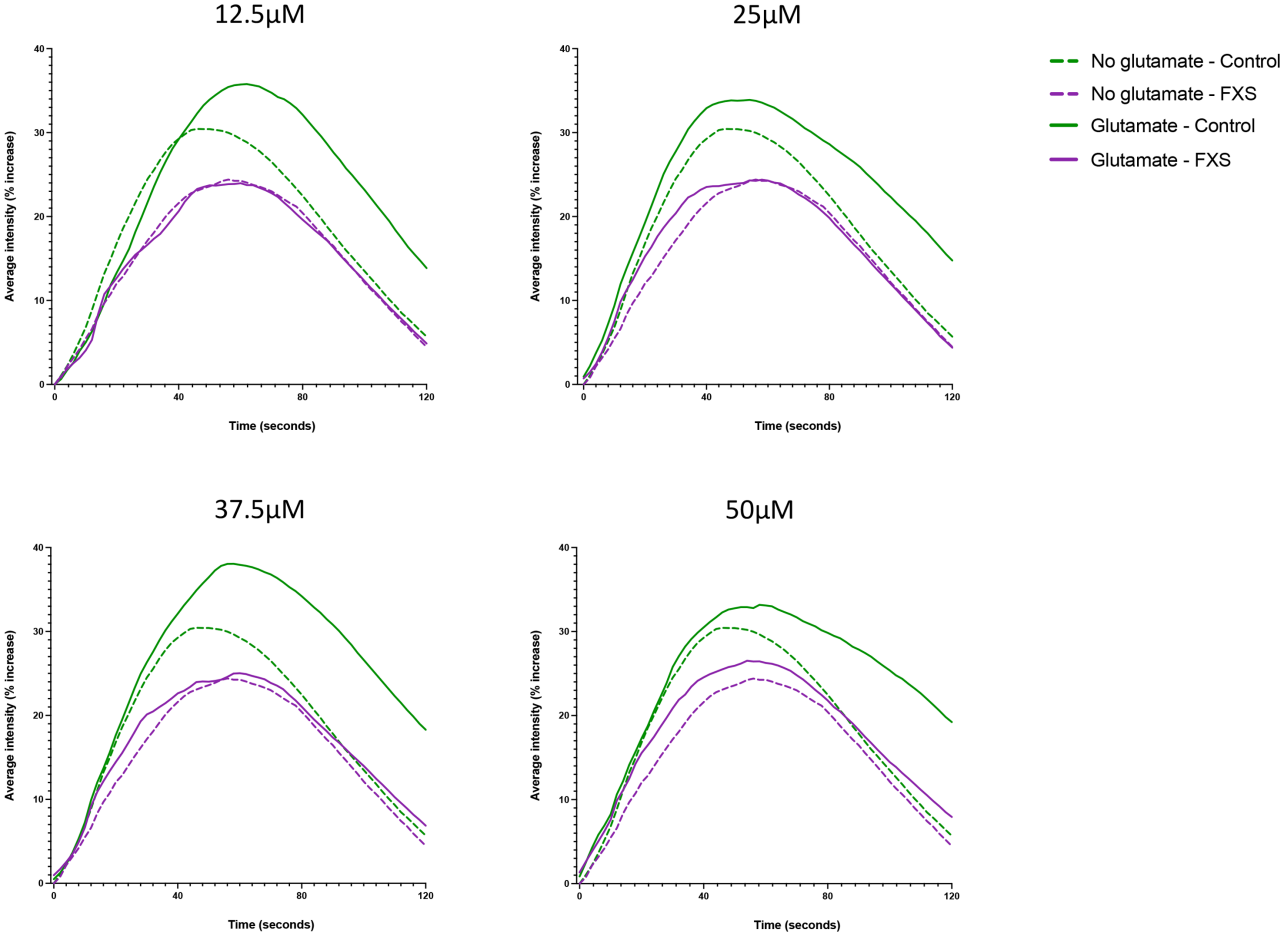
Representative Ca^2+^ responses to extracellular application of glutamate in hiDFP-derived neuronal cultures. Cal-520 AM fluorescence was measured as the percentage increase in average fluorescence intensity relative to baseline fluorescence at time 0 (ΔF/F_0_) in Control and FXS hiDFP-derived neurons. The percentage increase in average fluorescence intensity was measured for 120 s in the presence of no glutamate, 12.5 μM, 25 μM, 37.5 μM and 50 μM glutamate; *n* = 100 cells derived from 2–3 technical replicates per cell line from control (*n* = 3); FXS- (*n* = 3) biological replicates.

Impaired glutamate receptor-mediated plasticity is linked to alterations in excitatory (E) and inhibitory (I) synaptic systems, resulting in an imbalance of E/I ratio and functional deficits in FXS (Bassell and Warren, 2008; Gibson et al., 2008; Harlow et al., 2010; Zhang et al., 2014). Therefore, the aim of the following study was to confirm the functional maturation of our hiDFP-derived neurons, and to determine whether FXS-hiDFP derived neurons exhibited functional impairments. The functional properties of the control and FXS hiDFP-derived neurons were assessed by glutamate-induced intracellular calcium fluctuations using the fluorogenic calcium-sensitive dye Cal-520 AM. The average fluorescence intensity increased in controls relative to the baseline (time 0 s) following stimulation with glutamate, with an average increase of 35.78% ± 3.09% (12.5 μM); 33.90% ± 5.45% (25 μM); 38.06% ± 4.86% (37.5 μM), and 33.18 ± 6.95% (50 μM) detected at a maximum response time of 61, 55, 57 and 59 s, respectively (Figure 8). This represents a 6.93% ± 3.21% (12.5 μM); 3.91% ± 2.67% (25 μM); 8.82% ± 2.86% (37.5 μM), and 3.94% ± 4.04% (50 μM) increase in the maximum average fluorescence intensity when corrected for background signal in the unstimulated (no glutamate) condition. In contrast, only a modest increase in calcium activity was detected in differentiated FXS-neurons following glutamate stimulation, with a 23.97% ± 15.20% (12.5 μM); 24.40% ± 15.30% (25 μM); 25.03% ± 13.19% (37.5 μM), and 26.52% ± 10.59% (50 μM) maximum average fluorescence intensity detected relative to baseline at a maximum response time of 59 s at 12.5 μM, 25 μM and 37.5 μM glutamate, and 55 s at 50 μM glutamate (Figure 8**)**. Following background correction, this represents a decrease in the maximum average fluorescence intensity of −0.29% ± 0.63% (12.5 μM) relative to baseline. Furthermore, only a modest increase was detected following stimulation with higher concentrations of glutamate, at 0.127% ± 0.94% (25 μM); 0.099% ± 1.68% (37.5 μM); and 2.11% ± 4.54% (50 μM) in FXS-neurons.

In summary, functional analysis indicates that hiDFP-derived neurons exhibit glutamate induced intracellular calcium properties consistent with glutamatergic receptor expression and excitatory neuron development. However, FXS- hiDFP-derived neurons exhibited impaired responsiveness to glutamate stimulation compared to controls, suggesting impaired functional maturation.

### 3.6. Investigating the morphological properties of FXS- hiDFP-derived cortical neurons

**Figure 9:**
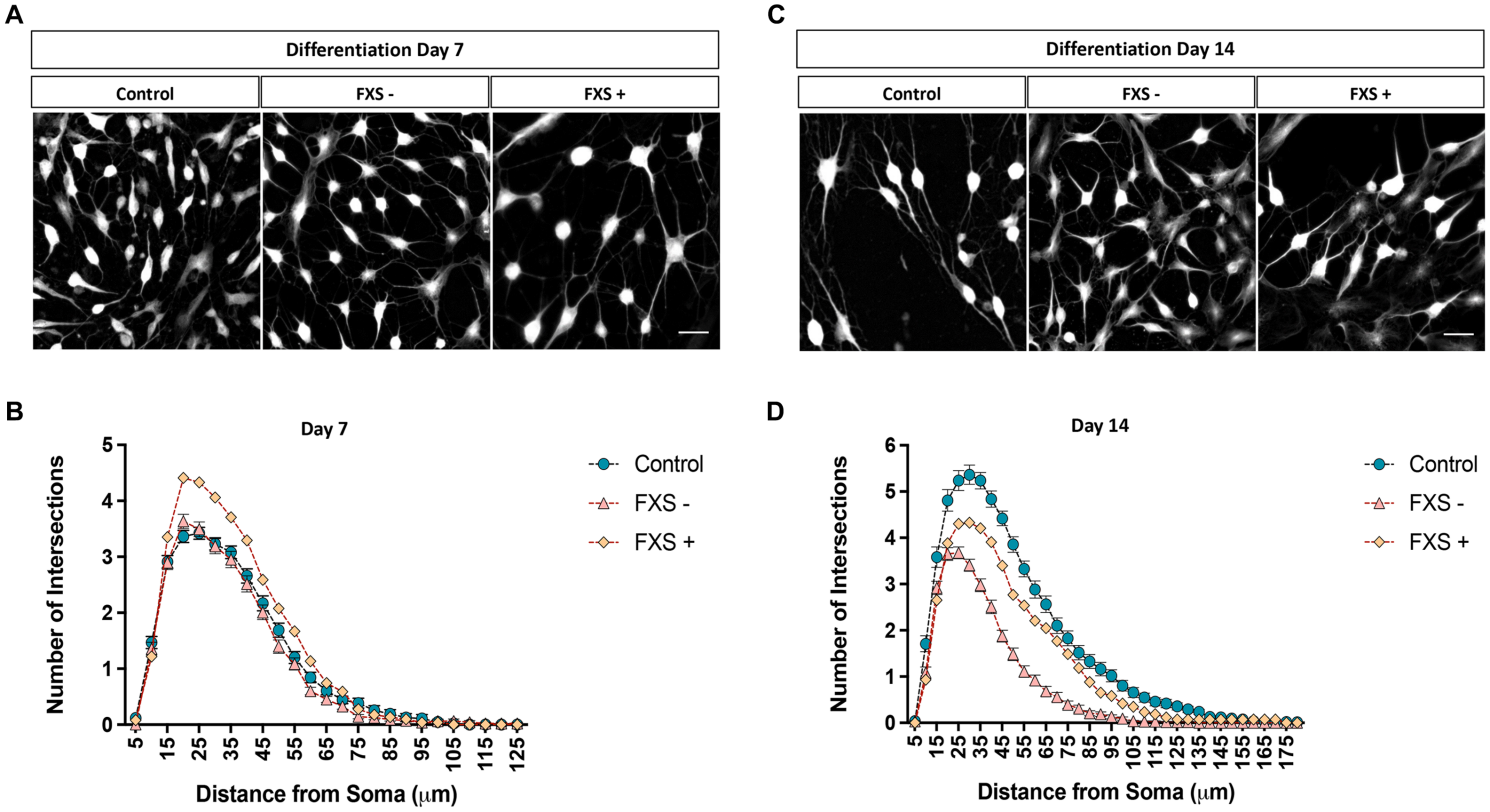
Complexity of the dendritic arbor of control and FXS hiDFP-derived neurons. **(A,C)** Representative images of TUJ1+ neurons after 7 **(A)** or 14 **(C)** days of differentiation converted to greyscale for semi-automated dendrite tracing. Scale = 50 μm. **(B,D)** Sholl plots representing the number of dendrites crossing concentric Sholl shells at 5 μm intervals from the cell soma in control and FXS hiDFP-derived neurons after 7 **(B)** or 14 **(D)** days of differentiation. Data represents the average ± SEM, control (*n* = 3); FXS- (*n* = 3) biological replicates from independent cell lines. Data also presented for FXS+ (*n* = 1).

**Figure 10:**
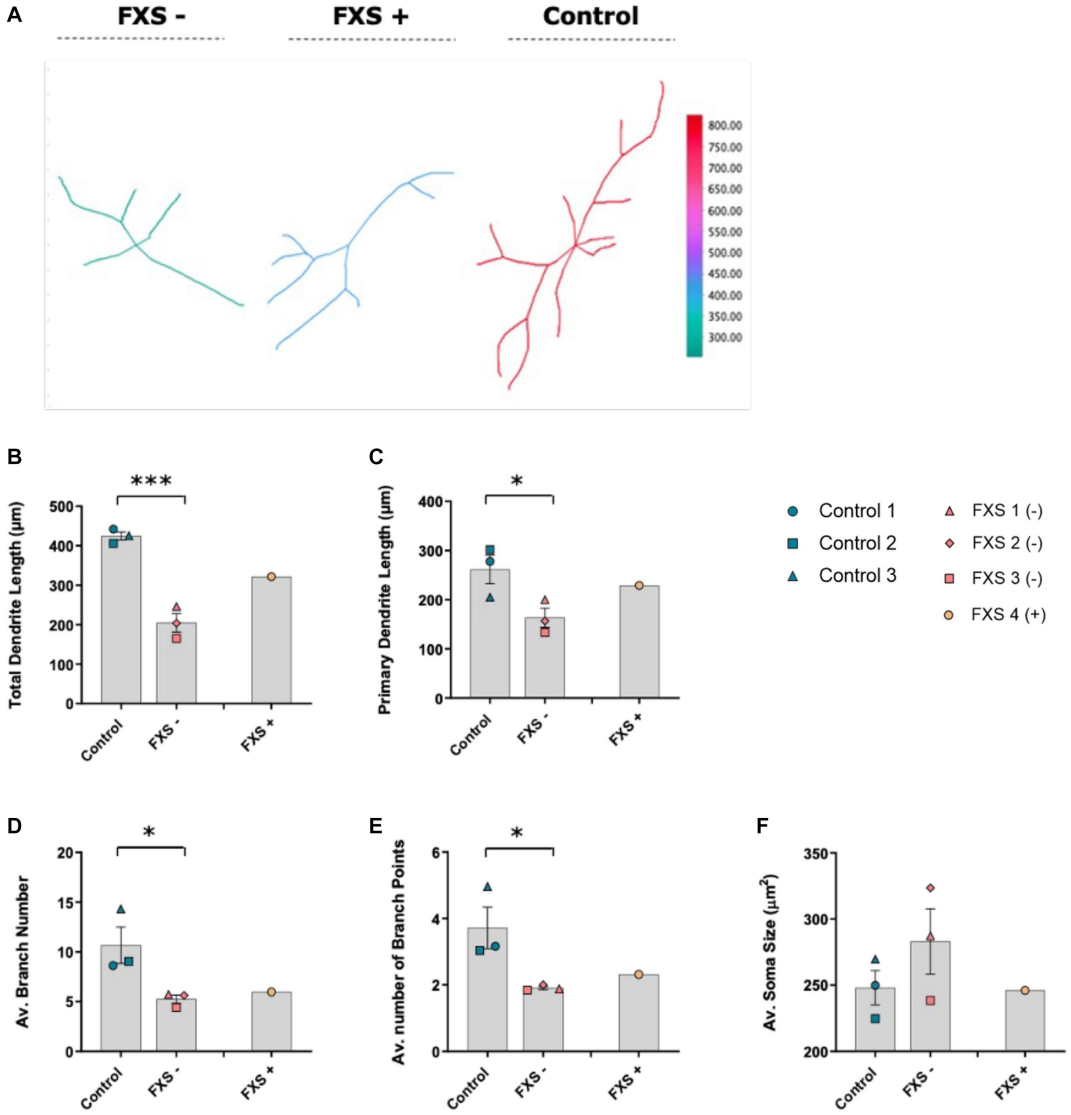
Morphological characteristics of control and FXS hiDFP-derived neurons at day 14 of differentiation. **(A)** Representative 2D plot of reconstructed neurons using the reconstruction plotter function in SNT. Dendritic arbors from manually traced hiDFP-derived Control, FXS-, and FXS+ neurons are automatically aligned, ranked and color-coded by total branch length as depicted in the color ramp legend. **(B–F)** Quantification of morphological parameters at day 14 of differentiation. All parameters quantified from a minimum of 40 neurons per cell line. Data represents the average ± SEM, control (*n* = 3); FXS- (*n* = 3) biological replicates from independent cell lines. Data also presented for FXS+ (*n* = 1). Statistical significance determined using an independent samples *t*-test, * *p* < 0.05, *** *p* < 0.001. FXS+ not included in statistical analysis.

The morphological properties of neurons are intrinsically linked to their functional maturation (Lefebvre et al., 2015). Dysregulated development of the dendritic arbor have been identified in *FMR1* KO or knock-down mouse models of FXS and human FXS-iPSC model systems. To determine the impact of impaired FMRP expression on dendritic maturation, the development of the dendritic arbor and cell soma were investigated in FXS affected hiDFP-derived neurons compared to controls. Sholl analysis was used to examine the number of dendrites intersecting concentric radii at 5 μm intervals from the cell soma. Total dendritic lengths, average branch number and branch points were automatically quantified per neuron from semi-automated tracing of TUJ1-immunopositive cells converted to greyscale (Figures 9, 10).

Following 7 days of differentiation, the peak in the average number of dendritic intersections was 20 μm from the cell soma. At this peak value, the average number of dendritic intersections for FXS- neurons was 3.63 ± 0.13, and 3.36 ± 0.11 for controls (Figures 9A,B). For the FXS+ cell line, a peak in the average number of dendritic intersections was identified at a 25 μm from the cell soma. At this peak value, the average number of dendritic intersections for FXS+ neurons was 4.33 ± 0.22 (Figures 9A,B).

After 14 days of differentiation, a peak in the average number of dendritic intersections was identified at a 30 μm radial distance from the cell soma for control lines. At this peak value, the average number of dendritic intersections for controls was 5.36 ± 0.21 and 3.67 ± 0.13 for FXS- (Figures 9C,D). For FXS- hiDFP-derived neurons, the peak in the average number of dendritic intersections was slightly closer to the cell soma, at a 25 μm distance. At this distance, the average number of dendritic intersections for FXS- neurons was 3.68 ± 0.13, and 5.24 ± 0.21 for controls (Figures 9C,D). For the FXS+ cell line, a peak in the average number of dendritic intersections was also identified at a 30 μm radial distance from the cell soma. At this peak value, the average number of dendritic intersections for FXS- neurons was 4.33 ± 0.22.

After 7 days of differentiation, no difference in cell soma size, total neurite length, primary dendrite length, average branch number or average number of branch points was observed between control and FXS- hiDFP-derived neurons (Supplementary Figure S4). However, following 14 days of differentiation, a significant difference in total dendrite length between control and FXS- hiDFP-derived neurons was determined, with FXS lines exhibiting a lower total dendritic length of 204.97 ± 9.86 μm, compared to controls, 424.58 ± 5.83 μm (Figure 10B; *t*(4) = 8.591, *p* = 0.001). A modest but significant difference in primary dendrite length was also observed between control and FXS- hiDFP-derived neurons at day 14 of differentiation, with FXS lines exhibiting a lower primary dendrite length of 163.43 ± 10.99 μm compared to controls at 266.03 ± 4.28 μm (Figure 10C; *t*(4) = 2.805, *p* = 0.049).

A significant difference in the average number of dendritic branches was detected between control and FXS- hiDFP-derived neurons at day 14 of differentiation, with FXS lines exhibiting a lower average branch number at 5.58 ± 0.04 compared to controls at 10.67 ± 0.07 (Figure 10D; t(4) = 2.772, *p* = 0.05). There was also a significant difference in the average number of dendritic branch points between control and FXS- hiDFP-derived neurons at day 14 of differentiation, with FXS lines exhibiting a reduced number of branch points at 1.9 ± 0.02 compared to controls at 3.72 ± 0.05 (Figure 10E; *t*(4) = 2.898, *p* = 0.044). No significant difference in cell soma size was determined between control and FXS-hiDF derived neurons at day 14 of differentiation (Figure 10F).

While the FXS+ line failed to acquire mature morphological characteristics comparable to controls over time, a moderate increase in total and primary dendritic length relative to the FXS- cohort was observed. While the significance of this could not be statistically determined from a single cell line, this trend suggests a possible correlation between FMRP expression and morphological maturation.

### 3.7. FXS- hiDFP-derived cortical neurons exhibit aberrant synaptic protein expression

FMRP plays a critical role in regulating the translation, stability and localization of mRNA targets encoding synaptic proteins required for functional synaptic development (Richter and Zhao, 2021). The phenotypic and functional impacts of FMRP loss on synaptogenesis are developmentally regulated and vary between model systems (Bagni and Zukin, 2019; Telias, 2019; Kang et al., 2021). Therefore, the aim of the following study was to assess synaptic development in FXS- hiDFP-derived cortical neurons compared to controls based on the density of pre- and post-synaptic protein expression.

**Figure 11:**
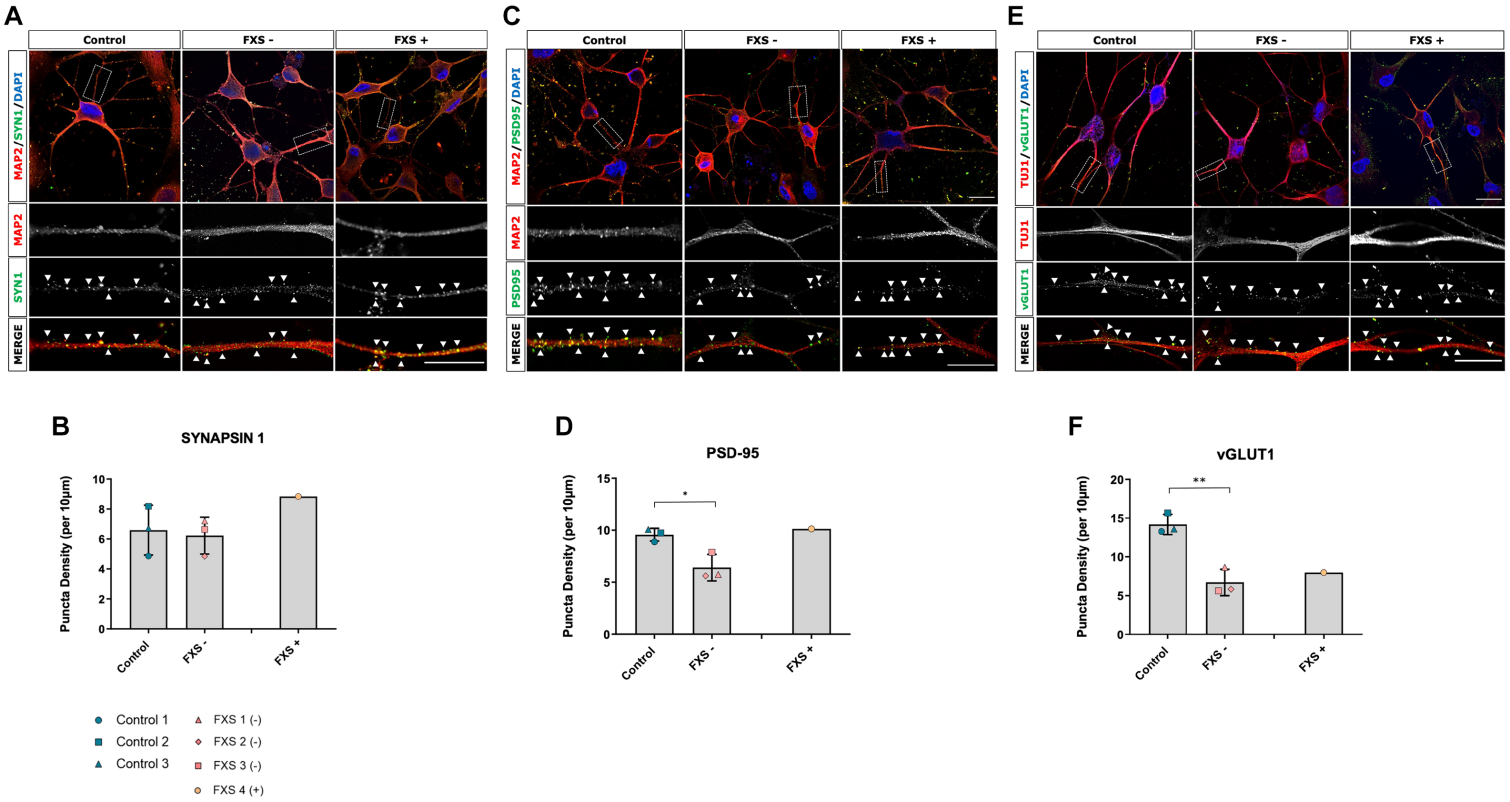
Synaptic protein expression in control and FXS hiDFP-derived neurons. **(A,C,E)** Representative images of Synapsin-1 **(A)**, PSD-95 **(C)** and vGLUT1 **(E)** protein colocalised with MAP2+ or TUJ1+ neurites. Scale = 20 μm. Arrow heads indicate positive puncta staining. **(B,D,F)** Quantification of Synapsin-1 **(B)**, PSD-95 **(D)** and vGLUT1 **(F)** measured as the density of protein puncta per 10 μm of MAP2+ or TUJ1+ neurite. Data represents the average ± SEM, control (*n* = 3); FXS- (*n* = 3) biological replicates from independent cell lines. Data also presented for FXS+ (*n* = 1). Statistical significance was determined using an independent samples *t*-test, * *p* < 0.05; ** *p* < 0.01. FXS+ not included in statistical analysis.

Following 14 days of cortical differentiation, no difference in the expression of the presynaptic protein Synapsin-1 was determined between control and FXS- hiDFP-derived neurons (Figures 11A,B). The presence of post-synaptic and glutamatergic pre-synaptic specialization was confirmed by the presence of PSD95 (Figure 11C) and vGLUT1 (Figure 11E), respectively, in both control and FXS hiDFP-derived neurons. There was a significant reduction in the expression of both PSD-95 and vGLUT1 on FXS- hiDFP-derived neurons compared to control neurons at day 14 of differentiation [Figure 11D; *t*(4) = 3.334, *p* = 0.029: Figure 11F; *t*(4) = 6.044, *p* = 0.004]. The reduction of PSD-95 and vGLUT1 protein expression observed in FXS- hiDFP-derived neurons corresponds to the lack of *DLG4* and *SLC17A7* gene expression in FXS- hiDFP-derived neurons compared to controls (Figure 6) and indicates that FXS- hiDFP-derived neurons exhibit impaired synaptic maturation.

## 4. Discussion

In FXS, the size and stability of the highly polymorphic CGG repeat tract in *FMR1* correlates with distinct phenotypic outcomes and severity. Carriers of the pre-mutation allele (55–200 CGG) for example, exhibit distinct phenotypes compared to full FXS (>200 CGG) affected individuals (Hagerman and Hagerman, 2004). Additionally, repeat size mosaicism within an individual is also linked to moderately increased levels of FMRP compared to full mutation alleles in males, and milder cognitive phenotypes (Baker et al., 2019). While increased repeat length confers susceptibility and elevated risk of FXS development, the methylation characteristics of the *FMR1* allele are highly predictive of FXS presentation. Methylation mosaicism for example has been linked to improved clinical outcomes in FXS (Pretto et al., 2014), while unmethylated full mutation alleles (UFM) in male carriers exhibit a reduction in FXS phenotype severity (Hagerman et al., 1994; Loesch et al., 2012) but are susceptible to the late onset-neurodegenerative disorder FXTAS (Hwang et al., 2016). The development of modelling strategies that recapitulate the diverse characteristics of the *FMR1* locus as it occurs in humans are required to better understand the relationship between genotype, epigenotype and phenotype throughout neuronal development. Such model systems will better facilitate our understanding of the mechanisms, timing, and impact of gene silencing throughout development.

This study represents the first reported derivation of FXS-affected cortical neurons following cmRNA-based direct reprogramming of patient fibroblasts to dorsal forebrain precursors. Methylation of the promoter is a key molecular hallmark of FXS in human tissue and an important modifier of phenotype severity. These effects are mediated by methylation mediated silencing of the *FMR1* gene and subsequently loss of FMRP, an important regulatory protein in neurodevelopment. Preservation of the methylation signature in the 5’upstream *FMR1* promoter in our study indicates that *SOX2/PAX6* cmRNA-mediated reprogramming and cortical differentiation did not induce epigenetic instability in either control or FXS cell lines. These findings also lend support to the hypothesis that correlations between the epigenotype and phenotype identified are representative of FXS, rather than artefacts of an unstable methylation signature introduced by the reprogramming mechanism or culture conditions. Interestingly, we identified one FXS cell line that exhibited *FMR1* expression and a promoter methylation signature consistent with a mosaic status. While unable to undertake statistical analysis, the mosaic FXS+ line provided insight into the effect of partial FMRP expression on neurogenesis. In summary, transcriptional (*FMR1*), epigenetic (% methylation), and phenotypic (FMRP) characterization of FXS hiDFPs and derived neurons confirmed that our model recapitulates the molecular hallmarks of FXS.

To confirm whether our *SOX2/PAX6* cmRNA direct reprogramming strategy could promote the generation of FXS-affected dorsal forebrain precursor cells, the expression of relevant lineage markers were assessed. Increased expression of genes required for specification of a dorsal telencephalic precursor fate were identified in both control and FXS- hiDFPs, including *FOXG1, NGN2, TBR2, TBR1* and *CTIP2.* This is also consistent with decreased expression of the ventral forebrain marker *DLX2*. The pan-precursor marker Nestin was also highly expressed across all hiDFPs cohorts. These findings were reinforced by protein expression analysis confirming expression of the phenotypic markers NGN2, FOXG1, TBR2, indicating dorsal forebrain precursor development. While no significant difference in the expression of the proliferative marker Ki67, or neural precursor markers, NGN2, TBR2 were detected, a significant increase in FOXG1 expression was identified in FXS- hiDFPs. FOXG1 is an important regulator of cell cycle progression and is an established ASD candidate gene (Mariani et al., 2015). Differential expression of this key developmental regulator may be indicative of dysregulated cell cycle progression of FXS- hiDFPs towards a neuronal lineage.

Following reprogramming, we wanted to confirm whether an enriched population of glutamatergic neurons could be generated from *SOX2/PAX6* cmRNA derived dorsal forebrain precursors. Additionally, differential expression of genes regulating neuronal fate specification, neuronal differentiation, cytoskeletal and axonal guidance, and synaptic signalling have been reported in FXS affected neurons (Halevy et al., 2015; Ferron, 2016; Lu et al., 2016; Danesi et al., 2018; Utami et al., 2020; Raj et al., 2021). Therefore, the current study investigated whether differential expression of key neuronal and synaptic genes could be identified in FXS compared to control hiDFP-derived neurons. Gene expression analysis following 14 days of differentiation indicated that FXS- hiDFP-derived neurons exhibited hallmarks of impaired neuronal development and maturation with a low fold-change increase of immature (*TUBB3*) and mature (*MAP2*) neuronal markers. Similarly, decreased expression of mature neurons markers *NSE* and *MAP-T* were observed. In FXS+ hiDFP-derived neurons, expression of mature neuronal markers was either low or decreased, suggesting partial FMRP expression was insufficient to mitigate impaired maturation at the transcriptional level.

Cortical neuron development was confirmed by increased expression of cortical layer specific neuronal markers in control and FXS hiDFPs following differentiation. Increased expression of the layer II-III specific markers *BRN2* and *CUX1* in control hiDFP-derived neurons is indicative of upper layer neuron development (McEvilly et al., 2002; Sugitani et al., 2002; Bulchand et al., 2003; Espuny-Camacho et al., 2013). Additionally, expression of *TBR1* (layer VI) and *CTIP2* (layer V) also indicate the presence of deep layer neurons (Hevner et al., 2001; Espuny-Camacho et al., 2013). Elevated expression of *BRN2* was similarly detected in FXS- hiDFP-derived neurons, although no change in *CUX1* expression relative to hiDFPs was detected. Although patterns of cortical lamination could not be assessed in our 2D *in vitro* system, impaired *CUX1* expression in layers II/III of *FMR1* KO mice have been linked to impaired migration of early born neurons to superficial layers of the cortex during corticogenesis (Lee et al., 2019). Impaired *CUX1* expression in FXS- hiDFP-derived neurons may therefore indicate impaired migration and neural positioning competence. Additionally, *CUX1* expression has been shown to regulate dendrite morphology and dendritic spine maturation in upper layer cortical neurons in *CUX1* null mice (Cubelos et al., 2010). However, these findings have yet to be replicated in human derived FXS neurons. The critical role of *CUX1* in dendrite branching and synapse formation may also indicate impaired competence for proper dendrite elaboration and synapse formation in *CUX1* deficient FXS- hiDFP-derived neurons. In addition to late born upper layer neuron markers, an increase in *TBR1* expression and lack of *CTIP2* expression suggests deep layer neuron specification in FXS- hiDFP-derived neurons. The expression of cortical layer markers in FXS+ hiDFP-derived neurons were comparable to controls, with the exception of *TBR1*, which was highly upregulated. Together, these results suggest a mixed population of deep and upper layer neurons of a glutamatergic lineage in both control and FXS hiDFP-derived neurons, with evidence of impaired neuronal maturation in FXS- but not FXS+ neurons. This contrasts with the findings of Kang et al. (2021), in which a bias towards deep layer neuron specification in FXS iPSC derived neurons was observed. Interestingly, FXS iPSC- derived deep layer neurons exhibited accelerated maturation, increased synapse formation and network hyperexcitability (Kang et al., 2021), contrary to our own findings.

Impaired cognitive function in FXS patients is associated with an imbalance of excitatory and inhibitory synapses (Gibson et al., 2008; Nelson and Valakh, 2015). Efforts to understand the mechanisms underpinning the imbalance of excitation and inhibition during early neural circuit formation in neurodevelopmental disorders, including FXS, are ongoing (Bagni and Zukin, 2019). Impaired synaptic homeostasis is linked to the loss of the activity-dependent functions of FMRP during postnatal periods of synaptic refinement (Tessier and Broadie, 2008; Pfeiffer et al., 2010). Therefore, the signalling events mediating cortical hyperexcitability and impaired inhibitory activity in FXS are thought to be largely mediated by post-transcriptional events and may not necessarily be reflected at the transcriptional level (Antar et al., 2006; Bassell and Warren, 2008). Nonetheless, elevated expression of the *SLC17A7* gene during neuronal development is prerequisite for the molecular assembly of functional excitatory synapses (Wojcik et al., 2004), and its upregulation in control hiDFP-derived neurons is indicative of glutamatergic lineage acquisition (Takamori et al., 2000). Although the expression of *SLC17A7* was confirmed in FXS- and FXS+ hiDFP-derived neurons, this was greatly reduced compared to controls. We also investigated expression of the synaptic protein encoding genes, *SYN1* (synapsin-1), and *DLG4* (PSD-95), which have established roles in regulating excitatory glutamatergic synapse function together with *SLC17A7* (vGLUT1). While both *SYN1* and *DLG4* were expressed in control neurons, neither gene exceeded a 2-fold change threshold for expression in FXS- hiDFP-derived neurons following 14 days of differentiation.

FMRP modifies L-type voltage-gated calcium channels and sodium-/calcium-activated potassium channels through regulating mRNA expression at the plasma membrane or binding ion channels directly (Brown et al., 2001; Darnell et al., 2011; Deng et al., 2013; Ferron et al., 2014) *CACNA1C* encodes the alpha-1 subunit of L-type voltage-dependent calcium channels (Ca𝙑.2,) and is predominantly expressed in glutamatergic neurons of the forebrain. Ca𝙑.2 has been shown to play a central role in activating calcium-mediated signalling pathways necessary for the differentiation of cortical neurons, dendritic growth, and plasticity, and is a known candidate susceptibility gene for a range of related disorders characterized by impaired cognitive function (Paşca et al., 2011). Similarly, hyperpolarization-activated cyclic nucleotide-gated channels (HCN) play fundamental roles in regulating neuronal excitability, and impaired homeostatic H-channel plasticity has been observed in *FMR1* deficient mice (Brager et al., 2012). While the expression of *CACNA1C* was upregulated in both control and FXS hiDFP-derived neurons, fold change expression was lower in FXS- hiDFP-derived neurons. Additionally, while *HCN2* was expressed in controls, fold-change expression remained low in FXS- hiDFP-derived neurons. Low *CACNA1C* expression coupled with a lack of *HCN2* expression above a 2-fold threshold in FXS- hiDFP-derived neurons may indicate impaired development of functional synapses compared to controls. Further studies examining whether alterations in the wider synaptic milieu exist at the protein level are required. The functional consequences of dysregulated protein expression at the synapse would provide clarity on the impact of the reduced gene expression of key synaptic markers identified in our study.

Following 14 days of differentiation, we detected a significant reduction in the yield of FXS- hiDFP-derived neurons compared to controls. This is consistent with previous reports demonstrating that FMRP deficiency during neural precursor development leads to reduced neuronal production and maturation (Luo et al., 2010; Guo et al., 2011). However, other studies investigating neurogenesis in FXS human pluripotent stem cells have reported both impaired (Boland et al., 2017), enhanced (Castren et al., 2005) and no change (Zhang et al., 2018) in neuronal yield. Despite the decrease in the neuronal yield of FXS- hiDFPs following differentiation, neuronal subtype specification to a glutamatergic neuronal fate was not affected. Interestingly, while no difference in the proportion of neuronal cells expressing a glutamatergic phenotype was observed, we detected a significant increase in the percentage of S100β + cells in FXS- hiDFP-derived cultures compared to controls following 14 days of differentiation. An increase in the proportion of S100β + cells following differentiation of FXS hiDFPs was surprising given the enhanced expression of *FOXG1* observed following reprogramming. *FOXG1* has been shown to drive a developmental bias towards neuronal over glial progenitor development (Brancaccio et al., 2010), as well as promote neurogenic competence in mouse derived astrocytes following forced expression (Ma et al., 2018). However, the mechanism by which elevated *FOXG1* might affect this neurogenic bias in the context of FMRP loss has not been reported. Reductions in neuronal yield and maturation in FXS studies have been attributed to impaired neurogenesis due to a shift in fate specification towards a glial cell lineage (Luo et al., 2010; Sunamura et al., 2018; Brighi et al., 2021). The mechanisms by which FMRP loss during neurogenesis may alter the ratio of neurons to astrocytes, and when this occurs remains unclear. However, these studies support the importance of FMRP in regulating neuronal lineage specification as well as survival and maturation.

Neuronal function and network development are hallmarks of proper neuron development. We therefore sought to confirm the generation of functional neurons and to assess differential functional properties in FXS- hiDFP derived neurons compared to controls. Elevations in intracellular calcium are an indirect result of action potential-driven membrane depolarization. We therefore utilized calcium imaging as an unbiased population level proxy for neuronal activity. Live-cell calcium imaging in control hiDFP-derived neurons confirmed intracellular calcium responses consistent with functional glutamatergic receptor expression. Notably, FXS- hiDFP-derived neurons exhibited dampened calcium responses following glutamate stimulation across a range of concentrations compared to controls. While impaired functional maturation is consistent with impaired development of the dendritic arbor and synaptic protein expression, our findings contrast with reports of increased calcium conductance and hyperexcitability associated with FMRP loss in both rodent and human cell models of FXS (Contractor et al., 2015; Achuta et al., 2018; Danesi et al., 2018; Brighi et al., 2021). Follow-up studies are therefore required to determine the mechanism of the impaired glutamate-induced calcium response observed in FXS- hiDFP-derived cortical glutamatergic neurons.

Alterations in the local repertoire and abundance of synaptic proteins in the postsynaptic compartment are linked to modification of the activity-dependent intracellular calcium response mediated by glutamatergic receptors. Expression of the scaffolding protein PSD-95, for example, modifies glutamate transmission through alterations in NMDA and AMPA-receptor recruitment, stability, and function in the postsynaptic compartment (El-Husseini et al., 2000; Ehrlich and Malinow, 2004; Chen et al., 2015), and is associated with cognitive phenotypes in a range of neurodevelopmental disorders (Tsai et al., 2012; Lelieveld et al., 2016). Determining whether the reduced PSD-95 protein abundance observed in our model is associated with altered subunit specific NMDA and AMPA receptor localization and function may clarify the functional impact of impaired synaptic protein expression on calcium flux in our model.

FMRP has established functions in the nuclear export of putative FMRP mRNA targets, regulating localized activity-dependent translation of proteins important for the integrity of cytoskeletal architecture and synaptic maturation. Given proper elaboration of the dendritic arbor is required for neuronal network formation and function (Lefebvre et al., 2015), the morphological properties of FXS hiDFP-derived neurons were assessed. In addition to a reduction in neuronal yield, morphological abnormalities in FXS- hiDFP-derived neurons consistent with impaired structural maturation were identified. Morphological analysis revealed a reduction in dendritic outgrowth and complexity as reflected in a reduction in total and primary dendrite lengths, average number of branches, and branch points in FXS- affected neurons. Interestingly, this difference was not observed relative to controls during early neurogenesis at day 7, suggesting that while FXS- hiDFP-derived neurons fail to acquire a properly ramified dendritic arbor consistent with mature neuron development, initial dendritic outgrowth remains unaffected. The absence of morphological deficits in immature FXS- hiDFP-derived neurons at day 7 of differentiation, followed by delayed morphological development by day 14 suggest defective neuronal maturation not impaired differentiation of hiDFP to neurons. The reduction in neurite length and dendritic arborization identified in FXS- hiDFP-derived neurons is consistent with phenotypes observed in both neonatal and adult *FMR1* KO or knock-down mouse models of FXS (Castren et al., 2005; Shen et al., 2019; Yau et al., 2019; Lannom et al., 2021) and human FXS patient derived iPSCs (Sheridan et al., 2011; Doers et al., 2014; Achuta et al., 2018; Utami et al., 2020). Additionally, neurospheres derived from human fetal postmortem tissue and human iPSC-derived neurons have demonstrated altered differentiation characterized by fewer and reduced neurite extensions (Castren et al., 2005; Doers et al., 2014). These findings however, conflict with a later report by Boland et al. (2017) in which an increase in the neurite length of *FMR1* deficient iPSC-derived neocortical glutamatergic neurons was observed at day 12 of differentiation. Interestingly, Boland et al. (2017) observed the effect of *FMR1* loss on neurite morphology was transient, with no net difference in phenotype observed by day 15 of differentiation relative to controls. Morphological assessments in the study by Boland and colleagues were limited to early neurogenesis. However, downregulated expression of key genes regulating axonal guidance and neurite growth at day 80 suggest impaired neuronal and synaptic maturation may also occur during later stages of neuronal development (Boland et al., 2017).

In addition to the cell intrinsic mechanisms effected by neuronal FMRP loss, the morphological abnormalities identified in FXS- hiDFP-derived neurons may be attributable to an increased population of S100β + astrocytes. Jacobs et al. (2010) demonstrated that hippocampal neurons from wild type mice exhibited delayed dendritic arborization and impaired synaptic maturation following co-culture with FMR1−/− astrocytes. Additionally, aberrant dendritic morphologies and reduced pre- and post-synaptic proteins were restored in hippocampal neurons from *FMR1* KO mice following coculture with WT astrocytes (Jacobs et al., 2010). More recent studies have indicated that alterations in astrocyte secreted signals such as Thrombospodin-1 (TSP-1; Cheng et al., 2016), as well as aberrant BMP signaling (Caldwell et al., 2022) in *FMR1*-deficent astrocytes results in impaired neurite outgrowth.

Proper elaboration and maturation of dendritic morphology is linked to synaptic protein expression (Vessey and Karra, 2007) and is a key determinant of synaptic function (Wong and Ghosh, 2002). Therefore, the expression of synaptic proteins required for maturation of the glutamatergic synapse in hiDFP-derived neurons was examined. Furthermore, FMRP regulates the stability and localized translation of putative mRNA targets encoding synaptic proteins necessary for dendritic spine maturation and synaptic plasticity (Cruz-Martín et al., 2010; Pfeiffer et al., 2010; Darnell et al., 2011; Deng and Klyachko, 2021). FMRP loss is therefore implicated in the aberrant synaptic signaling and network hyperexcitability thought to underpin impaired cognitive function in FXS (Contractor et al., 2015). The current study therefore sought to determine whether key synaptic proteins were differentially expressed in FXS- hiDFP derived neurons. The expression of Synapsin-1, PSD95 and vGLUT1 positive puncta were indicative of pre- and postsynaptic glutamatergic specialization in both control and FXS hiDFP-derived neurons. However, the density of PSD95 and vGLUT1 puncta was significantly lower in FXS- neurons following 14 days of differentiation compared to controls. PSD95 is a key synaptic scaffolding protein that regulates the molecular organization of the protein enriched region of the postsynaptic membrane of excitatory neurons (Sheng and Kim, 2011). It has also been shown to regulate the developmentally appropriate pruning of dendritic spines and thus synaptic strength throughout development (Nikonenko et al., 2008; Zhang and Lisman, 2012; Cane et al., 2014). The observation that PSD95 expression was lower in FXS- hiDFP-derived neurons was surprising given the canonical role of FMRP in inhibiting the translation of its putative mRNA targets. Specifically, FMRP loss has been linked to enhanced association of target mRNA with translating polyribosomes (Muddashetty et al., 2007) and impaired degradation of PSD95 at the synapse (Tsai et al., 2012). In contrast, a significant decrease in PSD95 mRNA and protein expression due to loss of FMRP-mediated mRNA stability was detected by Zalfa et al. (2007) in *FMR1* KO mice. Interestingly, differential expression of PSD95 was only detected in the hippocampus and cerebellum and not cortical neurons of *FMR1* KO mice. This is consistent with the findings of Todd et al. (2003) who observed only a transient increase in PSD95 expression in the cortical neurons of *FMR1* KO mice compared to WT (Todd et al., 2003).

As changes in vGLUT1 expression are known to modify the strength of excitatory synaptic signaling (Daniels, 2004; Zhang et al., 2019) the observed reduction in the density of vGLUT1 puncta may suggest impaired excitatory synapse development in FXS- neurons compared to control neurons. However, reduced expression of vGLUT1 in FXS- neurons was unexpected given the reported role of FMRP in negatively regulating the translation of synaptic mRNA and reducing synapse number to regulate network activity (Pfeiffer and Huber, 2007). This is supported by a recent report by Brighi and colleagues who observed an increase in the co-localization of vGLUT1+/PSD95+ puncta during early development in FXS hiPSCs with a concomitant increase in spontaneous network activity (Brighi et al., 2021). Interestingly, this phenotype was found to be transient. Similarly, an aberrant increase in synaptic development as determined by an increase in the density of Synapsin-1+/PSD95+ synaptic boutons and enhanced neuronal excitability was detected in FXS hiPSCs (Kang et al., 2021). Interestingly, evidence of increased or unchanged expression of vGLUT1 containing synapses in the neocortex of *FMR1* KO mice was shown to be dependent on layer specific neuronal identity with no change observed in cortical neurons (Wang et al., 2014). Collectively, the reduced density of PSD95 and vGLUT1 puncta may be indicative of impaired or delayed development of mature functional synapses in FXS- hiDFP-derived neurons. This is consistent with the observed impairment in expression of mature neuronal markers and evidence of immature dendritic arbor development. However, validating and quantifying the co-localization of postsynaptic (PSD95) and presynaptic (Synapsin-1, vGLUT1) proteins as a representative feature of functional synapses within FXS- hiDFP-derived neurons is necessary to confirm whether loss of protein expression correlates with a loss of synapse density in our study.

Although differential expression of key markers of neuronal maturation and function were identified in our model system, low sample size is a key limitation of our study. The number of independent biological replicates reported in cell reprogramming studies is typically low, reinforcing the practical limitations of scaling such strategies to align with the large sample sizes often required to power such analyses (Germain and Testa, 2017). Multiple clones derived from 1 to 2 individual donors are frequently utilized in iPSC-based studies to improve sample size. This approach often artificially reduces group mean variability when multiple clones from the same individual are treated as statistically independent (Germain and Testa, 2017). Our direct reprogramming method is not susceptible to the genomic variability that arises through clonal expansion (Abyzov, 2012; Young et al., 2012) and the extended culture times (Mayshar et al., 2010) observed in iPSC-based approaches. The donor-to-donor variability identified in this study is thought to reflect the true heterogeneity of independent biological replicates included. Although scalability is limited by the availability of relevant FXS affected donor tissue, increased sample sizes are required to validate our findings in future studies and to capture smaller effect sizes.

In conclusion, this study represents the first reported derivation of FXS-affected cortical glutamatergic neurons following reprogramming of patient fibroblasts directly to dorsal forebrain precursors. Importantly, the FXS-associated phenotypes detected in this study were identified in human neuronal cells that recapitulate the molecular hallmarks of the FXS mutation as it occurs in FXS-affected individuals. Differential expression of mature neuronal markers suggests impaired neuronal development and maturation in FXS- hiDFPs derived neurons compared to controls. This is supported by the reduced yield of FXS- hiDFP-derived neurons and increase in FXS- affected astrocytes following 14 days of differentiation. Additionally, FXS- hiDFP-derived cortical neurons exhibited dendritic growth and arborization deficits characterized by reduced neurite length and reduced neurite branching consistent with impaired neuronal maturation. The significant decrease in the density of pre- and post- synaptic proteins in FXS- hiDFP-derived neurons suggests impaired excitatory synapse development. While consistent with an immature neuronal phenotype, further co-localization and functional maturation analysis is required to determine whether this finding is consistent with aberrant synapse formation and network function reported in both *FMR1* KO animal and patient derived FXS-iPSCs. The differential phenotypic outcomes reported in this study and others are likely to result from differences in the developmental stage and relative maturity of neurons derived by various modelling strategies, and likely indicate regional and neuronal cell subtype specific effects of FMRP loss, which are often poorly defined. Whereas the aberrant morphological phenotypes observed in human postmortem tissue captures the accumulated impact of FMRP loss, both established *FMR1* KO mouse models and emerging cell reprogramming based strategies indicate the importance of the stage dependent effects of FMRP loss on phenotypic outcomes. The functional effects of transient morphological phenotypes to the neurodevelopmental progression of FXS reinforces the importance of defining the relative maturity of derived neuronal populations to allow meaningful comparisons between studies, as well as the need to refine the temporal resolution of developmental modelling strategies to better capture and understand the effects of intermittent phenotypes throughout development. Despite discrepancies in phenotypic outcomes reported, collectively these studies indicate that FMRP is strongly implicated in the morphogenesis of the dendritic arbor and synaptic maturation. The magnitude and direction of these effects appear to be contingent on developmental stage, neuronal subtype and modelling strategy employed.

## Data availability statement

The original contributions presented in the study are included in the article/Supplementary material, further inquiries can be directed to the corresponding author.

## Ethics statement

Ethical approval was not required for the studies on humans in accordance with the local legislation and institutional requirements because only commercially available established cell lines were used.

## Author contributions

BC: Conceptualization, Funding acquisition, Project administration, Resources, Supervision, Validation, Writing – original draft, Writing – review & editing. NE: Formal analysis, Investigation, Methodology, Resources, Writing – original draft, Writing – review & editing. CC: Formal analysis, Investigation, Methodology, Validation, Writing – review & editing. AM-C: Investigation, Methodology, Writing – review & editing.

## Funding

This work was supported by the Neurological Foundation of New Zealand and the Kate Edger Educational Charitable Trust (KEECT).

## Conflict of interest

The authors declare that the research was conducted in the absence of any commercial or financial relationships that could be construed as a potential conflict of interest.

## Publisher’s note

All claims expressed in this article are solely those of the authors and do not necessarily represent those of their affiliated organizations, or those of the publisher, the editors and the reviewers. Any product that may be evaluated in this article, or claim that may be made by its manufacturer, is not guaranteed or endorsed by the publisher.
