# Supplementary material for: Directly reprogrammed fragile X syndrome dorsal forebrain precursor cells generate cortical neurons exhibiting impaired neuronal maturation
Source: Front Cell Neurosci. 2023 Sep 21;17:1254412. doi: 10.3389/fncel.2023.1254412 (PMC10552551; doi:10.3389/fncel.2023.1254412)
Supplement: Supplementary file 1 [file Image_1.pdf]

**Solution**	Ingredient	Quantity
**0.1 M PB (pH 7.2)**	$Na_2H_2PO_4.H_2O$ (Scharlau)	3.84 g
	$Na_2H_2PO_4$ (Scharlau)	10.2 g
	MilliQ H_2O	Final Volume 1 L
**4% PFA (pH 7.2 – 7.4)**	PFA (Sigma-Aldrich)	20 g
Filter sterilised, stored at -20°C	0.1 M PB (60°C)	250 mL
	0.1 M PB (4°C)	250 mL
**10X PBS**	KH_2PO_4 (Scharlau)	2 g
	Na_2HPO_4 (Scharlau)	11.5g
	KCl (Scharlau)	2g
	NaCl (Scharlau)	80 g
	MilliQ H_2O	Final Volume 1 L
**1X PBS (pH 7.4)***	10X PBS	100 mL
	MilliQ H_2O	900 mL
**1X PBS-T**		
**(0.5% Triton-X-100)**	Triton-X-100	2 mL
**(pH 7.4)**	10X PBS	100 mL
	MilliQ H2O	898 mL

*Autoclaved and stored at room temperature

Supplementary Figure 1. Solutions prepared for immunocytochemistry.
