# Supplementary material for: Directly reprogrammed fragile X syndrome dorsal forebrain precursor cells generate cortical neurons exhibiting impaired neuronal maturation
Source: Front Cell Neurosci. 2023 Sep 21;17:1254412. doi: 10.3389/fncel.2023.1254412 (PMC10552551; doi:10.3389/fncel.2023.1254412)
Supplement: Supplementary file 2 [file Image_2.pdf]

Assay ID: ADS1451-FS1	
**Genomic Target Sequence**	**Bisulfite converted Target Sequence**
cgttctggccctcgcgaggcagtgcgacctgtcaccgccc<br>ttcagccttcccgccctccaccaag	YGTTTTGGTTTTYGYGAGGTAGTGY<br>GATTTGTTATYGTTTTTTAGTTTTT<br>TYGTTTTTTATTAAG

Assay ID: ADS1451-FS2	
**Genomic Target Sequence**	**Bisulfite converted Target Sequence**
cccgcgcacgcccggcccgcgcgtctgtctttcgacccgg<br>caccccggccggttcccagcagcgcgcatgcgcgcgctc<br>ccaggccacttgaagagagag	TTYGYGTAYGTTYGGTTYGYGYGT<br>TTGTTTTTYGATTYGGTATTTYGGT<br>YGGTTTTTAGTAGYGYGTATGYGY<br>GYGTTTTTAGGTTATTTGAAGAGAG<br>AG

Supplementary figure 2. Target sequence of the EpigenDX human FMR1 gene promoter assays. (Ensembl Gene ID: ENSG00000102081).
