# Supplementary material for: Directly reprogrammed fragile X syndrome dorsal forebrain precursor cells generate cortical neurons exhibiting impaired neuronal maturation
Source: Front Cell Neurosci. 2023 Sep 21;17:1254412. doi: 10.3389/fncel.2023.1254412 (PMC10552551; doi:10.3389/fncel.2023.1254412)
Supplement: Supplementary file 3 [file Image_3.pdf]

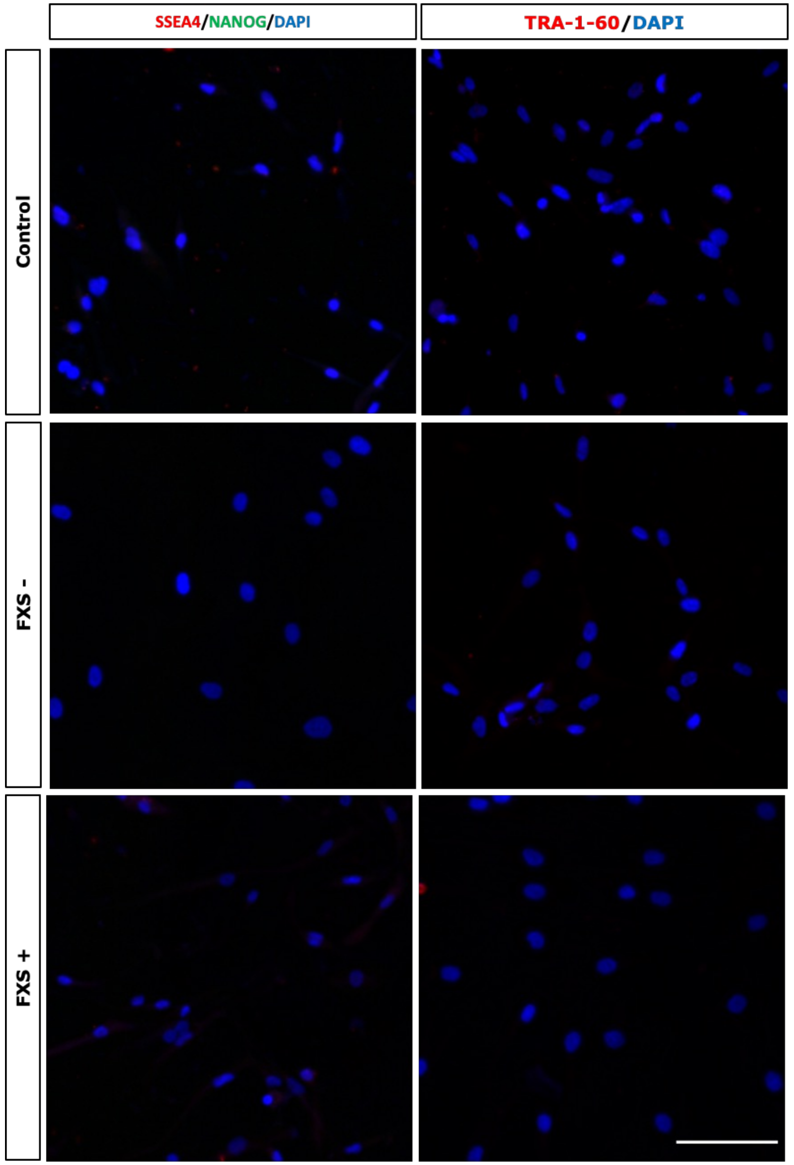

Supplementary Figure 3. Control and FXS hiDFPs do not express pluripotency markers following reprogramming. Representative images of hiDFPs demonstrating a lack of SSEA4, NANOG and TRA-1-60 expression following 21 days of reprogramming. Scale bar = 60 µm.
