# Supplementary material for: Directly reprogrammed fragile X syndrome dorsal forebrain precursor cells generate cortical neurons exhibiting impaired neuronal maturation
Source: Front Cell Neurosci. 2023 Sep 21;17:1254412. doi: 10.3389/fncel.2023.1254412 (PMC10552551; doi:10.3389/fncel.2023.1254412)
Supplement: Supplementary file 4 [file Image_4.pdf]

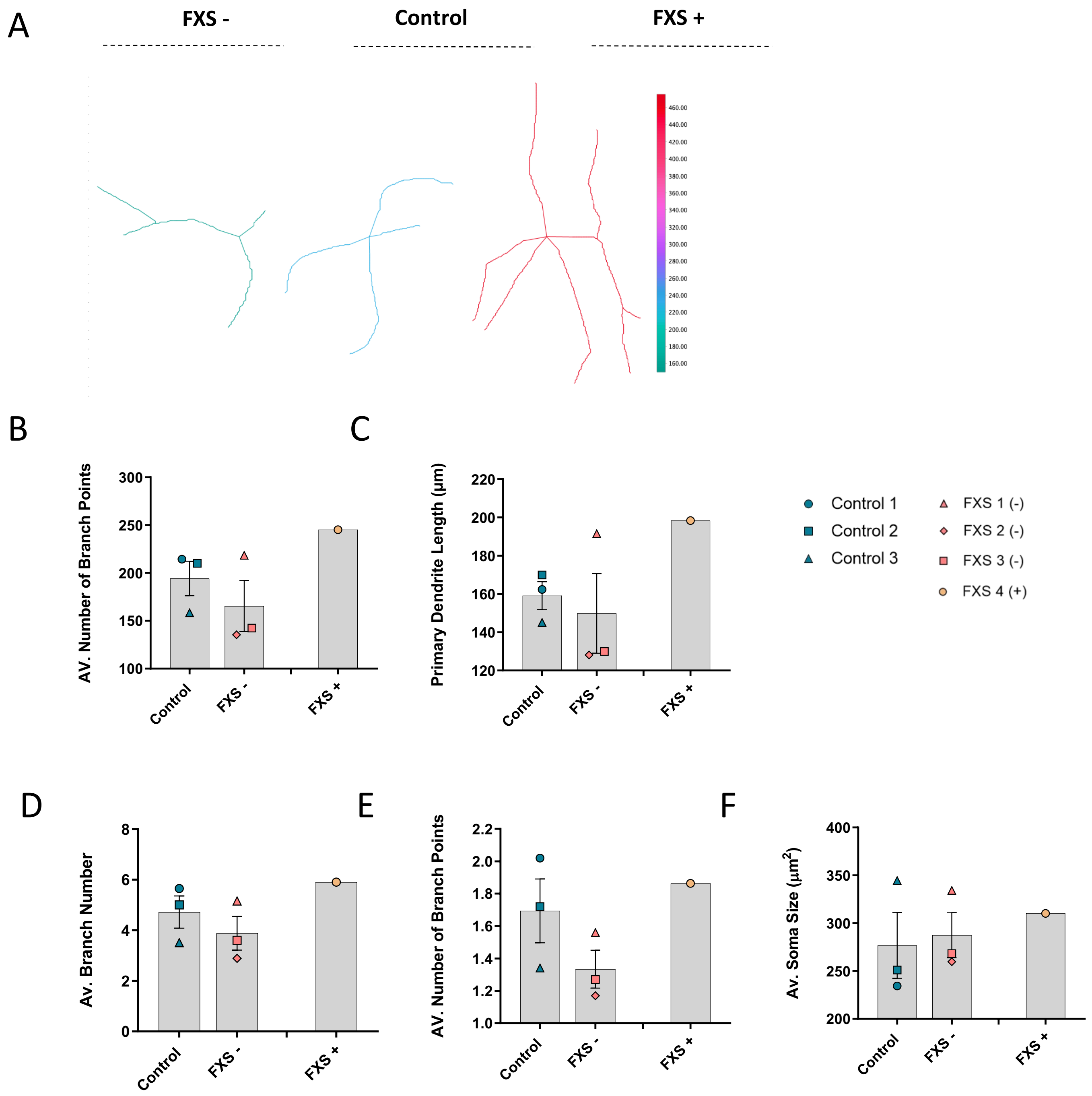

Supplementary Figure 4. Morphological characteristics of control and FXS hiDFP-derived neurons at day 7 of differentiation. (A) Representative 2D plot of reconstructed neurons using the reconstruction plotter function in SNT. Dendritic arbors from manually traced hiDFP-derived Control, FXS-, and FXS+ neurons are automatically aligned, ranked and color-coded by total branch length as depicted in the color ramp legend. (B - F) Quantification of morphological parameters at day 7 of differentiation. All parameters quantified from a minimum of 40 neurons per cell line. Data represents the average ± SEM, Control (n= 3); FXS- (n=3) biological replicates from independent cell lines. Data also presented for FXS+ (n=1).
